# mTORC1 Promotes SARS-CoV-2 Replication and Attenuates Type I Interferon Responses in the Alveolar Epithelium: A Computational and Experimental Study

**DOI:** 10.1007/s11538-026-01701-8

**Published:** 2026-09-02

**Authors:** Achyudhan R. Kutuva, Henrique AL Ribeiro, Felipe T. Lima, Matthew A. Schaller, Borna Mehrad, Reinhard Laubenbacher

**Affiliations:** 1 Division of Pulmonary, Critical Care and Sleep Medicine, University of Florida, Gainesville, 32611 FL USA; 2 Department of Computational and Systems Biology, School of Medicine, University of Pittsburgh, Pittsburgh, 15261 PA USA; 3 Department of Immunology, School of Medicine, University of Pittsburgh, Pittsburgh, 15261 PA USA

**Keywords:** SARS-CoV-2, MTORC1, IFN, Boolean network

## Abstract

The mechanistic target of rapamycin complex 1 (mTORC1) has been implicated in coronavirus pathogenesis, yet its precise role in shaping antiviral defenses in pneumocytes remains unresolved. This study combines *ex vivo* human lung tissue assays with a literature-curated, logic-based (Boolean) signaling model to study how mTORC1 influences SARS-CoV-2 replication and type-I interferon (IFN) responses. Our in-vitro data showed that pharmacologic inhibition of mTORC1 with sirolimus was associated with reduced viral replication and increased IFN-β expression across donor samples. To interpret these data, we constructed a network integrating inflammation, stress, apoptosis, and RIG-I–IFN signaling, and evaluated four mechanistic hypotheses for explaining our experiments in which mTORC1 either promotes replication, inhibits IFN, both, or neither. The model was constructed according to the best evidence in the literature and evaluated against 56 independent protein/phosphoprotein readouts from 26 studies not used for model construction; the model achieved high qualitative accuracy overall (F1 ≳0.75) and on live-virus experiments (F1 ≳0.9). We found that the hypotheses where mTORC1 promotes viral replication, inhibits IFN, or both were able to qualitatively reproduce the empirical data, *i.e*., mTORC1 inhibition by sirolimus leads to lower viral replication and higher IFN expression. We queried the robustness of those predictions through a systematic edge knock-in/knockout screen generating 6,328 network variants per scenario (25,312 total). We found that the scenario where mTORC1 enhances viral replication is the more plausible, due to parsimony (this mechanism is also observed in other viral infections), higher robustness (more variants still reproduce the result), and stronger inhibition that is more likely to be observed in biological experiments with low statistical power. This scenario supports the idea that the mTORC1 effects on IFN expression are indirectly mediated by viral replication itself.

**Supplementary Information:**

The online version contains supplementary material available at https://doi.org/10.1007/s11538-026-01701-8.

## Introduction

Understanding how host cells respond to viral infections at the intracellular level remains a central challenge in infectious disease biology. In particular, SARS-CoV-2, the virus responsible for COVID-19, has demonstrated a complex interplay with host signaling pathways that modulate viral replication, immune activation, and cell fate decisions. Among these pathways, the mechanistic target of rapamycin complex 1 (mTORC1) has emerged as a critical regulator of viral pathogenesis, influencing both viral protein synthesis and immune signaling cascades (Basile et al. 2022; Fattahi et al. 2022). However, the precise role of mTORC1 in shaping the antiviral response—especially its impact on type-I interferon (IFN) expression—remains incompletely understood.

Recent experimental studies in *ex vivo* human lung cultures have demonstrated that SARS-CoV-2 infection activates mTORC1 in type I pneumocytes (Basile et al. 2022). At the same time, *in vitro* data from a tissue culture system, presented here, show that pharmacological inhibition of mTORC1 with Sirolimus reduces viral replication and enhances IFN-β expression. While increase of viral replication by mTORC1 has been shown for other viruses (see Fattahi et al. (2022); Basile et al. (2022)), to the best of our knowledge, our results show for the first time an increase of IFN-β expression during SARS-CoV-2 infection led by mTORC1 inhibition. These findings suggest a dual role for mTORC1 in promoting viral replication and suppressing antiviral signaling. Yet, mechanistic clarity is lacking: it is unclear whether both effects are directly mediated by mTORC1 or if one arises as a secondary consequence of the other.

Boolean networks provide a well-suited framework for investigating intracellular signaling when detailed kinetic parameters are unavailable or highly uncertain. By encoding regulatory interactions as qualitative logic rules derived from the literature, Boolean models enable the integration of heterogeneous experimental findings into a coherent mechanistic structure and allow systematic exploration of competing hypotheses about pathway interactions. This approach has been used widely in the literature to model intracellular signaling in cells such as macrophages (Palma et al. 2018; Marku et al. 2020), T-cells (Saez-Rodriguez et al. 2007), and others (Brandon et al. 2015). At the same time, Boolean networks have important limitations. They lack an explicit representation of physical time and do not capture concentration-dependent kinetics, which constrains their ability to make quantitative predictions. Despite these limitations, Boolean networks offer a transparent and computationally efficient means of probing complex immune–viral signaling landscapes and generating mechanistic insights that can guide further experimental and quantitative modeling efforts.

We developed a mechanistic computational model to explore mTORC1’s role in SARS-CoV-2 infection. We employed a qualitative network (QN) modeling approach, a logic-based framework that enables the simulation of complex regulatory systems using literature-curated associations and limited quantitative data (Schaub et al. 2007). The model integrates key pathways implicated in viral sensing, inflammation, metabolism, and cell death, including NF-κB, PI3K/AKT, p53, and AMPK—alongside mTORC1 and its upstream and downstream regulators (Fattahi et al. 2022; Bramante CT, Beckman KB, Mehta T, Karger AB, Odde DJ, Tignanelli CJ, Buse JB, Johnson DM, Watson RHB, Daniel JJ, Liebovitz DM, Nicklas JM, Cohen K, Puskarich MA, Belani HK, Siegel LK, Klatt NR, Anderson B, Hartman KM, Rao V, Hagen AA, Patel B, Fenno SL, Avula N, Reddy NV, Erickson SM, Fricton RD, Lee S, Griffiths G, Pullen MF, Thompson JL, Sherwood NE, Murray TA, Rose MR, Boulware DR, Huling JD, Team C-OS 2024; Wang et al. 2023; Mukherjee 2022). Using this framework, we formulated and tested four mechanistic hypotheses regarding mTORC1’s influence on viral replication and type I IFN signaling.

This work includes two sets of data: the literature used to build and evaluate the model, and our own data generated in-house. Our in-house data suggested novel hypotheses concerning the relation between mTORC1, viral replication, and type-I IFN expression. The goal of the mechanistic modeling was to explore which of these hypotheses best reproduces our in-house data. Distinguishing among these hypotheses can affect treatment choice, especially for treatments that affect targets downstream of mTORC1. We further used the model to identify putative regulatory motifs and potential drug targets that modulate the antiviral response.

By combining computational modeling with experimental data, this work contributes to a growing body of research that leverages systems biology approaches to dissect host-pathogen interactions. The resulting insights have implications for both the basic understanding of SARS-CoV-2 pathogenesis and the rational design of therapeutic strategies targeting host pathways (Ribeiro et al. 2023; Chowdhury et al. 2022; Adhikari et al. 2022; Fan et al. 2025).

## Materials and Methods

### Experimental Methods

#### Recruitment, sample collection, and processing

We obtained lung tissue samples from lung donors who had undergone lung resection surgery or from oversized allografts that underwent volume reduction before transplant surgery. In cases of lung resection for nodules, samples far from the site of pathology were utilized. Pleural tissue and staple lines were dissected away, and the lung was sectioned into approximately 0.5 g pieces, divided between the wells of a 24-well plate, and manually disintegrated with surgical scissors into samples with a mean diameter of 0.91 mm (range 0.40–1.5 mm). Each well was resuspended in 1.2 mL of commercial DMSO-free cryopreservation medium (CryoSOfree, C9249; MilliporeSigma), divided into 200 μL aliquots, and transferred to cryotubes containing 800 μL of additional cryopreservation media. Samples were frozen at –$80^{\circ }$C overnight, then moved to vapor-phase liquid nitrogen storage.

#### Viral culture

The SARS-CoV-2 strain UF1 was propagated in Vero E6 cell line (ATCC CRL1586) in advanced DMEM (12491015; Gibco, Thermo Fisher Scientific) supplemented with 10% HyClone Defined low antibody, heat-inactivated, γ-irradiated fetal bovine serum (FBS; SH30070.03IR2540; Cytiva); 1% l-alanine and l-glutamine supplement (GlutaMAX, 35050061; Gibco, Thermo Fisher Scientific); and 1% penicillin/streptomycin mixture (17-602E; BioWhittaker, Lonza), at $37^{\circ }$C in 5% $CO_2$ in 75 $cm^2$ flasks.

We used cell culture with approximately 80% confluency in the monolayer to inoculate the virus. The cell culture media were removed from flasks and replaced with 3 mL of fresh media; We added 1 mL of viral stock (containing $10^6$ PFU - Plaque-Forming Unit) to each flask; and the flask was incubated at $37^{\circ }$C in 5% $CO_2$ for 1 hour, with manual rocking every 15 minutes.

We observed the flasks daily for development of cytopathogenic effect (CPE) and harvested after 96 hours of incubation when CPE had reached at least 95% of cells and approximately 25% cell detachment. The media were removed and the contents centrifuged at 2000g for 10 minutes at room temperature. The supernatant was then stored at –$80^{\circ }$C overnight in 1 mL aliquots. Viral titer of each batch was determined via 0.9% methylcellulose plaque assay with crystal violet staining 7 days after inoculation, after formalin fixation. The plaque assay protocol was modified from a previous study. Lednicky et al. (2020); Miller et al. (1950)

#### Tissue culture, infection, and drug treatment

The viral stock was thawed and diluted to a final concentration of $10^4$ PFU/well, in lung culture media, composed of 2x Lonza Bronchial Epithelial 1 Growth Medium, phenol-red-free DMEM (21063-029, Gibco, Thermo Fisher Scientific), 2% FBS (HyClone Defined low antibody, heat-inactivated, γ-irradiated fetal bovine serum; SH30070.03IR2540; Cytiva), and 1.5 μL retinol. Cryopreserved lung samples were thawed for 5 minutes in the $37^{\circ }$C water bath, then transferred to a 15 mL tube containing 10 mL DPBS using 1000 μL wide-bore pipette tips and centrifuged at 200g for 1 minute. The supernatant was aspirated, and samples were resuspended in 1 mL lung culture media and plated in flat-bottom 96-well cell culture microplates in lung culture media with the testing compounds.

Tissues were infected with the specified PFU in 200 μL of media for 24 hours. The inoculated plates were incubated at $37^{\circ }$C in 5% $CO_2$ for 24 hours, after which 100μl of media was removed and replaced with 100μl of fresh media containing the testing compound, and cultured for another 24 h. We tested the sirolimus (A10782; Adooq Bioscience) at a concentration of 20 nM to study its effect on viral replication (Olajide et al. 2021; Anastassopoulou et al. 2020). To calculate the percentage inhibition for sirolimus, which is soluble in DMSO, samples were infected and treated with an equal volume of DMSO in the absence of drug to determine the maximum signal.

Biopsy sample concentrations were estimated by counting the number of tissue fragments in 20 μL on a microscope slide, and 20–40 biopsies in 20 μL were added to each virus-containing well. Each vial of biopsies provided sufficient tissue for approximately 10 wells at this concentration. Because this culture system does not allow enumeration of cells per tissue at the start of the experiment, viral inocula were quantified as PFU/mL, corresponding to a multiplicity of infection (MOI) of 0.01–0.1 in *in vitro* infection of human cells (42–44). Tissues were exposed to the specified PFU in 100 μL of medium and incubated for 24 hours at $37^{\circ }$C (or $33^{\circ }$C for HCoV-OC43 infections) in 5% $CO_2$.

#### Flow cytometry

Samples were resuspended in BEGM+DMEM with 125 ng/mL Liberase (LIBTM-RO, 5401119001; MilliporeSigma) and 50 U/mL DNase I (D5025; MilliporeSigma), agitated for 1 hour at $37^{\circ }$C, and then aspirated 30 times into a 1 mL syringe through an 18 G needle to form a single-cell suspension. Cells were centrifuged at 400g for 5 minutes at room temperature, washed twice in RPMI-1640, resuspended in flow cytometry buffer (1 mL PBS with 2% FBS and 1 mM EDTA), and filtered through 100 μm Nitex nylon mesh (57-103; Genesee Scientific).

Samples were washed and resuspended in 100 μL PBS and stained with a fixable viability dye (Zombie Aqua, 423101; BioLegend) for 10 minutes at room temperature, protected from light. Cells were then washed and resuspended in 100 μl buffer and stained with the following antibodies against surface cell markers: PE anti-human CD140a (BioLegend 323505; PE/Cyanine 7); anti-human CD326 (9C4 BioLegend 324222); Zombie Aqua Fixable Viability Kit (BioLegend 423101); Alexa Fluor 647 anti-human Podoplanin (BioLegend 916610); Alexa Fluor 700 anti-human CD45 (BioLegend 368513); APC/Cyanine7 anti-human CD206 (BioLegend 321120); APC anti-human HLA-DR (Beckman Coulter IM3635); Brilliant Violet 605 anti-human CD31 (BioLegend 303121); Brilliant Violet 785 anti-human CD3 (BioLegend 317330).

We stained the samples with the surface marker antibodies, which were added at a concentration of 0.5 μL per 100 μL, then incubated for 20 minutes at room temperature on the orbital shaker while protected from light. After incubation, the samples were washed twice with PBS. Resuspended samples were analyzed on a Cytoflex (Beckman-Coulter). Flow cytometry data were analyzed using FlowJo X (BD Biosciences).

#### Virus quantification

RNA was isolated using Zymo Direct-zol RNA MicroPrep kits according to the manufacturer’s instructions (11-330MB; Genesee Scientific) and quantified using a NanoDrop spectrophotometer (Thermo Fisher Scientific), and 50 ng was reverse-transcribed using iScript cDNA Synthesis Kit (1708891; Bio-Rad). Real-time PCR was performed using Applied Biosystems TaqMan Gene Expression Master Mix (4369016; Thermo Fisher Scientific). Nucleocapsid protein 1 was assessed using primer/probe mixes (2019-nCoV RUO Kit, 10006713), and a standard curve was generated using the 2019-nCoV_N_Positive Control (10006625; both from Integrated DNA Technologies).

We used the predeveloped primer/probe assays from Thermo Fisher Scientific (IFNB1 Hs01077958_s1). The $\Delta \Delta$ Ct was calculated using the 18S ribosomal RNA primer/probe set (4319413E; Thermo Fisher Scientific).

### Computational methods

#### Computational modeling of a pneumocyte infected with SARS-CoV-2

We used the method of Schaub et al. (2007) to construct the Boolean rules in the model. Activation of a node is given by the average of the activators minus the average of inhibitors, although traditional Boolean functions (*i.e.*, AND, OR, and NOT) are used in some cases (See Appendix B for more details).

We performed all model simulations using random asynchronous update orders. Therefore, the behavior of the model changed from simulation to simulation. We ran ensembles of 500 different update orders and computed the average (Figure S2). Our final results are in the range -1 to 1, and represent the difference between the stimulated simulation (Figure S2B) and resting simulation (Figure S2A). See Section B.4 for more details.

**Fig. 1:**
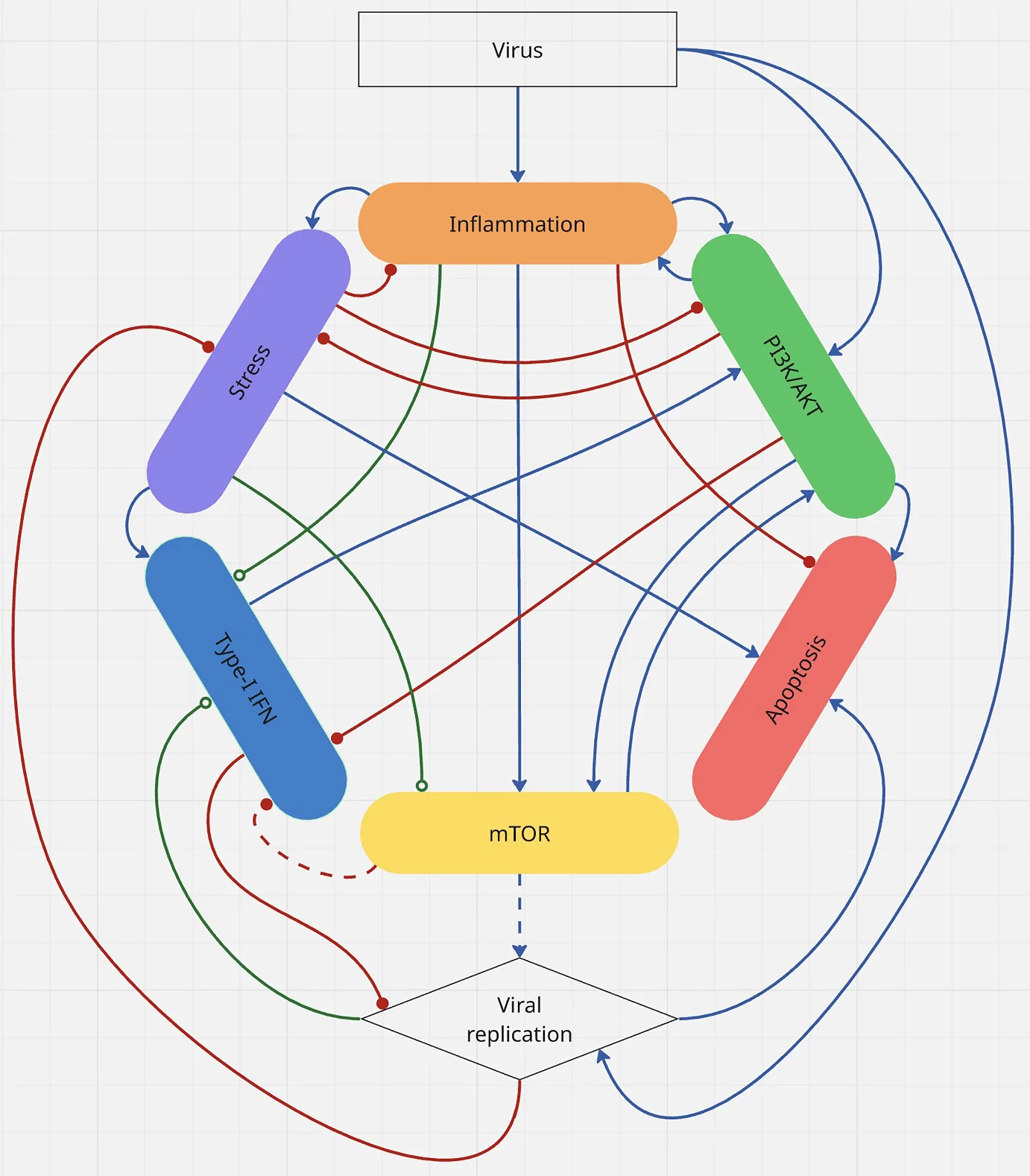
Map showing the relationship between the conceptual modules in the model (details below). Blue arrows indicate activation, red inhibition, green activation and inhibition (*i.e.*, at least one component of the module led to activation while another led to inhibition of the target module). Dashed arrows represent scenario-dependent activation or inhibition (see Section 2.2.2). The modules in the wiring diagram are: Inflammation: NF-κB, MAPK, and Angiotensin – Renin/Angiotensin was merged with inflammation in this figure for conciseness; PI3K/AKT pathways; Apoptosis pathway; mTOR pathway; Type I IFN: RIG-1, IRF3, IFN, STAT1, and ISG; Stress: AMPK, p53, and HIF1-α. Because each node in this wiring diagram summarizes a module with many genes and proteins, they can influence each other in different and opposing ways. For example, stress-related factor AMPK activates mTORC2 but inhibits mTORC1, both of which are in the mTOR pathway (Color figure online)

**Fig. 2:**
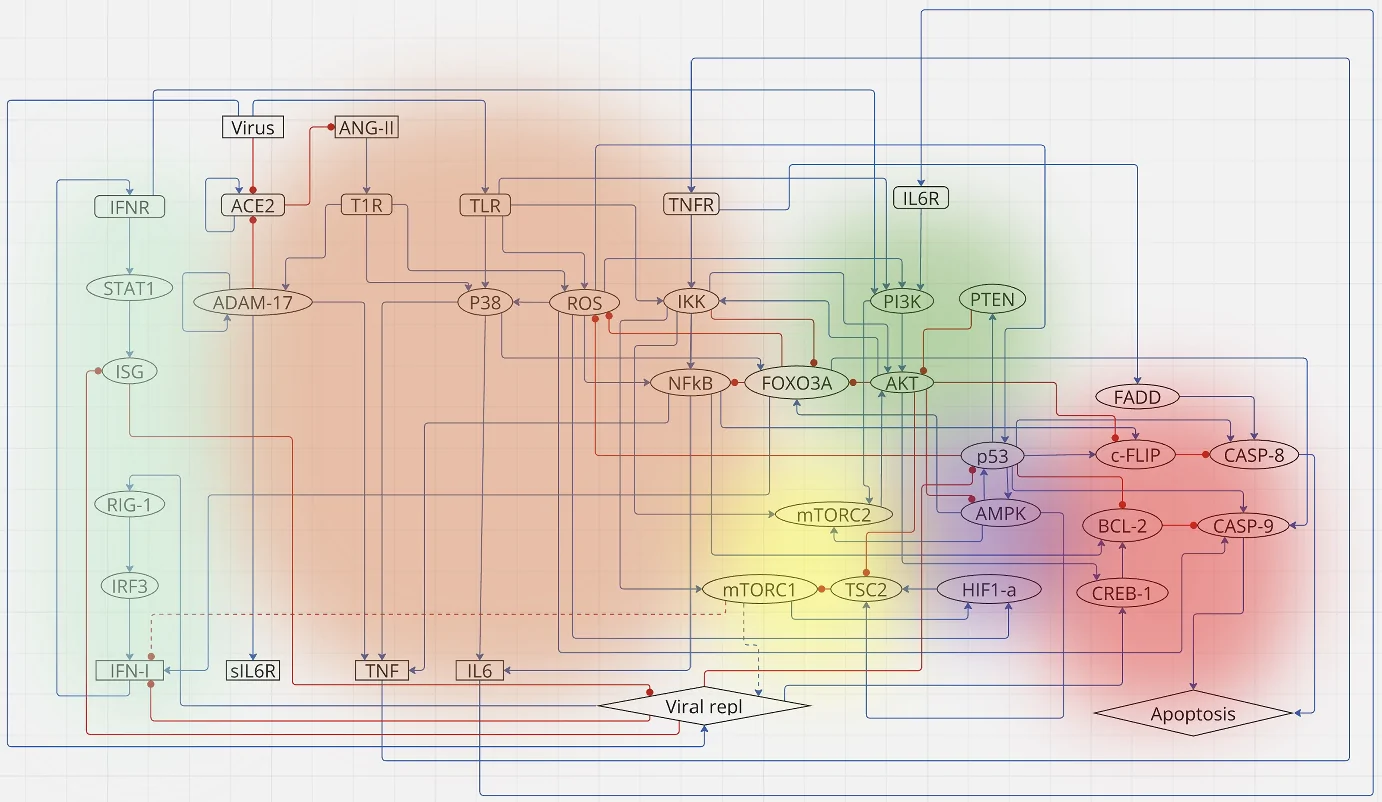
Wiring diagram of the model. Regular rectangles represent inputs/outputs, rectangles with rounded corners receptors, ellipses intracellular molecules, and diamonds phenotypes. Blue arrows are activation and red round arrows inhibition. The dashed arrows represent hypothesized connections that are modeled in some scenarios (see section 2.2.2), but not in others. The several modules are shown in different colors. Inflammation/angiotensin (brown), PI3K/AKT (green), mTOR (yellow), stress-related pathways - p53, AMPK, and HIF-1α (purple), apoptosis (red), type-I IFN production and response (light-bluish-green) (Color figure online)

The structure of the model is summarized in Fig. 1, a conceptual map containing the nine signaling pathways or pathway groups. While this abstraction is useful for visual clarity, it omits certain nuances present in the full model (Fig. 2), such as dual regulatory roles where a pathway can both activate and inhibit a target, depending on context. Figure 2 depicts the complete model, with all major signaling modules highlighted. Tables S2 and S3 show the complete set of equations and the literature justification.

We created an initial model with literature data, which is summarized in Table S2. This model was evaluated against new literature data not used to build it (see Section 2.2.4).

The most commonly infected cells are those from the respiratory tract, such as pneumocytes (Carcaterra and Caruso 2021; Jackson et al. 2022). The infection starts with the virus invading them by binding to the ACE2 receptor and shedding it Wang et al. (2022a). When ACE2 is shed, the body’s ability to convert Angiotensin-II into Angiotensin-1-7 declines, leading to the accumulation of Angiotensin-II, which triggers inflammation (Ni et al. 2020).

**The inflammation/Angiotensin pathways.** The inflammation pathways talk to all other modules in the model (Fig. 1). The virus triggers inflammation via TLR4, as well as Angiotensin-II (Mukherjee 2022; Wang et al. 2022a). The inflammation pathways connect with PI3K/AKT via IKK-$\alpha /\beta$. This kinase both activates and is activated by AKT (Dan et al. 2016; Zhao et al. 2017). IKK-$\alpha /\beta$ also offers a link with the mTOR family, where it can activate both mTORC1 and mTORC2 (Dan et al. 2016). On the other hand, the stress factor p53 is activated by ROS, and in turn, p53 inhibits ROS (Hu et al. 2010; Zhang et al. 2021).

Inflammation primarily exerts inhibitory effects on apoptosis. NF-κB induces anti-apoptotic factors such as c-FLIP and BCL-2, and IKK-$\alpha /\beta$ inhibits FOXO3A pro-apoptotic signaling (Hu et al. 2004; Sikder et al. 2014; Safa 2013). However, p38 has the opposite effect (Marzi et al. 2016), and TNF-α has pro-apoptotic activity through FADD-mediated caspase-8 induction (Weinlich and Green 2014).

FOXO3A enhances type I IFN production (Gimenes-Junior et al. 2019). Meanwhile, inflammation has a dual relationship with this transcription factor, with IKK-$\alpha /\beta$ inhibiting and p38 activating it Weinlich and Green (2014); Hu et al. (2004). Therefore, inflammation can both increase or decrease type I IFN expression, depending on the factors activated (Hu et al. 2004; Gimenes-Junior et al. 2019; Weinlich and Green 2014).

**The PI3K/AKT pathway** also connects with all the other modules (Figs. 1 and 2). Its entry node, PI3K, receives signals from different sources, such as IL-6, ROS, and TLR4 (inflammation), as well as type I IFN (Zegeye et al. 2018; Okoh et al. 2013; Koundouros and Poulogiannis 2018; Qiu et al. 2020; Lekshmi et al. 2023). On the other hand, PI3K/AKT activates inflammation by different routes. As mentioned, AKT both activates and is activated by IKK-$\alpha /\beta$ (Dan et al. 2016; Zhao et al. 2017). It also inhibits anti-inflammatory molecules and the pro-interferon transcription factor FOXO3A (Dan et al. 2016; Gimenes-Junior et al. 2019). PI3K/AKT and mTOR activate each other (Fig. 1). PI3K induces mTORC2, which in turn activates AKT (Basile et al. 2022; Dan et al. 2016). AKT activates mTORC1 via TSC2 inhibition (Feng and Levine 2010). That kinase also modulates apoptosis by both activating BCL-2 through CREB-1 and inhibiting c-FLIP (Zhao et al. 2017; Du and Montminy 1998; Lin et al. 2015). It inhibits stress-related kinase AMPK and is inhibited by the p53-induced protein PTEN (Zhao et al. 2017; Carracedo and Pandolfi 2008).

**The stress-related pathways** in the model include AMPK, p53, and HIF-1α. This module receives signals from inflammation, PI3K/AKT, mTOR pathways, as well as viral replication (Figs. 1 and 2). Both AMPK and HIF-1α inhibit mTORC1 by promoting TSC2 (Feng and Levine 2010; Brugarolas et al. 2004), while mTORC1 promotes HIF-1α (Brugarolas et al. 2004; Zhu et al. 2021). AMPK also promotes mTORC2 and the hub transcription factor FOXO3A (Kazyken et al. 2019; Zhao and Liu 2021). This factor led to anti-inflammatory, pro-apoptotic, and pro-interferon downstream signaling (Hu et al. 2004; Sikder et al. 2014; Safa 2013; Dan et al. 2016; Gimenes-Junior et al. 2019). p53 has mostly pro-apoptotic activity. Although it activates caspase-8 inhibitor, c-FLIP (Bartke et al. 2001), it can also activate caspase-8 directly (Liu et al. 2011), and inhibit BCL-2 (Wei et al. 2023). As noted above, p53 is activated by ROS (Zhang et al. 2021) and inhibited by viral replication (Ma-Lauer et al. 2016). It has anti-inflammatory and anti-PI3K/AKT activity (Hu et al. 2010; Jung et al. 2010).

**The mTOR pathway** is regulated by the inflammatory pathway PI3K/AKT, and stress modules; see (Figs. 1 and 2). Inflammation and PI3K/AKT are activators (Basile et al. 2022; Dan et al. 2016), while stress modules inhibit mTORC1 and activate mTORC2 (Feng and Levine 2010; Kazyken et al. 2019; Brugarolas et al. 2004). However, the big picture is somewhat more complex. mTORC2 leads to mTORC1 activation via AKT (Dan et al. 2016) and inflammation may inhibit mTORC1 via ROS-activated HIF-1α (Brugarolas et al. 2004; Zhu et al. 2021). mTORC1 has the hypothesized proviral replication and anti-interferon activity (see Section 2.2.2).

**The Apoptosis pathway** receives signals from inflammation, PI3K/AKT, and stress pathways (Figs. 1 and 2). The first two can be inhibitory (Safa 2013; Kucharczak et al. 2003; Du and Montminy 1998), although inflammation activates caspase-9 via ROS (Lin et al. 2015; Qian et al. 2022) and caspase-8 via TNF/FADD (Mohd Zawawi et al. 2023; Weinlich and Green 2014), while AKT inhibits c-FLIP (Skurk et al. 2004). Meanwhile, stress-related protein p53 is the strongest pro-apoptotic factor, excluding those in the apoptosis pathway itself (Wei et al. 2023; Xing et al. 2011).

**The type-I interferon (IFN) response** is triggered primarily by RIG-1, which senses the products of viral replication inside the cell, as shown in Figs. 1 and 2 (Yamada et al. 2021). However, FOXO3A is also reported to induce type-I IFN production (Gimenes-Junior et al. 2019). The secreted IFN binds to the IFN-receptor, triggering a group of genes called IFN-activated genes (ISG). Some of these decrease or even halt viral replication (Mohd Zawawi et al. 2023). The IFN receptor is also reported to trigger PI3K, which inhibits pro-interferon FOXO3A via AKT (Platanias 2005). While viral replication induces IFN transcription, some of those proteins also inhibit these pathways at several points (Tolomeo et al. 2022; Bösmüller et al. 2021).

**Finally, viral replication** is a convergence of several signals (Figs. 1 and 2). First, it is important to clarify the difference between a virus and viral replication. The virus represents the extracellular virion that binds to TLR4 and the ACE2 receptor, shedding it Wang et al. (2022a); Mukherjee (2022). But it also serves as a flag to indicate that the cell is infected, even if viral replication is off at that moment.

We coded the viral replication equation in two different ways depending on the modeled hypothesis (see Section 2.2.2). For some hypotheses, mTORC1 is not a part of the viral replication equation, while in others it is, namely those that model the hypothesis that the activation of this transcription factor boosts viral replication. We use the equation “$AVG(virus,\ AND(virus,\ mTORC1))\ -\ ISG$." to model that. The conjunction between virus and mTORC1 “(AND(virus,mTORC1))" is necessary to ensure that mTORC1 does not lead to viral replication in uninfected cells. Technically, mTORC1 does not lead to a boost in viral replication; instead, the lack of mTORC1 leads to a decline in viral production. However, those two mechanisms (boosted by mTORC1 or reduced by lack of mTORC1) are equivalent. They may only become inequivalent if we want to compare the exact average viral replication predicted by different hypotheses. In this work, we only make qualitative, not quantitative comparisons.

This model captures a wide range of documented regulatory interactions and reflects the complex interplay between viral sensing, inflammation, metabolism, and antiviral defense mechanisms. It provides a foundation for testing how specific pathways, particularly mTORC1, influence infection outcomes and interferon dynamics.

#### Model variants: the four hypotheses

The objective of this paper is to study the influence of mTORC1 on the infection. The literature (Basile et al. 2022; Fattahi et al. 2022) points out that mTORC1 is a promoter of viral infection, while our own data show that cells treated with Sirolimus, an inhibitor of mTORC1, had lower viral particles and higher Type I IFN expression (see Section 3.2). There are two possible mechanisms at work here: “(M1) mTORC1⊣IFN" and “(M2) $mTORC1\rightarrow viral\_replication$." Therefore, there are four resulting scenarios combining these:

1. mTORC1 increases viral replication. (NOT M1 AND M2)
2. mTORC1 increases viral replication AND inhibits type-I IFN expression. (M1 AND M2)
3. mTORC1 inhibits type-I IFN expression. (M1 AND NOT M2)
4. mTORC1 has no direct effect on viral replication nor type-I IFN expression. (NOT M1 AND NOT M2)

From this point, we will call these four variants Scenarios 1-4. However, in some cases, we will also refer to them as hypotheses 1-4 when we find it necessary to stress that these four scenarios are implementations of four hypotheses.

#### Running the Model

The model presented here is a Boolean network formulated using the formalism of Schaub et al. (2007) a detailed description of this framework is provided in Section B.1. Network updates are performed asynchronously with a stochastic update order, implying that individual simulation runs are not deterministic and may yield different outcomes across repetitions. For this reason, all analyses in this study are based on ensembles of network realizations rather than on single simulations. The ensemble methodology is described in detail in Section B.4; briefly, the results reported here correspond to ensemble averages, which are not restricted to binary values. Instead, ensemble outputs may take fractional values. In fact, as we will discuss shortly, the ensemble outputs range from -1 to 1.

Unlike ordinary differential equation models, Boolean networks do not possess an explicit notion of physical time. Instead, they are typically analyzed by running the dynamics until an attractor is reached and interpreting the system state at that attractor. The networks studied here do not exhibit classical Boolean attractors, as illustrated in Figure S2, but they do converge to well-defined steady states. Based on systematic convergence analysis (Section B.3), all simulations were run for 30 iterations, which was sufficient for steady-state behavior to emerge; in most cases, convergence occurred within fewer than 15 iterations. All results reported in this paper refer to the final iteration of each simulation. Strictly speaking, due to technical considerations described in Section B.4, results are computed as an aggregate over the last two iterations; for clarity, we refer to this simply as the “last iteration” throughout this subsection.

Biological experiments frequently compare a control condition with a perturbed condition—for example, baseline cell cultures versus cultures exposed to SARS-CoV-2. We explicitly mirror this experimental paradigm in our simulations. All reported results represent differences between the final steady-state values (last iteration) obtained under a “perturbed condition” simulation and those obtained under a corresponding “control” simulation. As a consequence, ensemble outputs span the interval [-1,1] rather than [0,1]. “Control” and “perturbed condition” simulations are always executed sequentially rather than in parallel, ensuring consistent baseline states (see Section B.4 for details).

#### Testing the model in an independent dataset

To test the four scenarios, we compared their respective predictions to a independent dataset not used to build the model comprised of 56 experiments from 26 publications. This dataset was made of human pneumocytes (A549 and Calu-3) infected with SARS-CoV-2 or incubated with SARS-CoV-2 S or N proteins, pneumocytes (A549) incubated in hypoxic conditions ( 1% oxygen), glucose starvation, TNF-α stimulation, Angiotensin-II stimulation, Sirolimus stimulation, and one experiment of LPS stimulation with HIF-1α KO cells. These papers report the expression of proteins and phosphoproteins and other outcomes, such as p-STAT1, p-AKT, p-4E-BP1 (mTORC1 proxy), Apoptosis (different forms of measurement), ACE2, ADAM17, ISG15, and others, in response to the stimuli mentioned previously (see Table S5).

With a few exceptions, all the literature data we used refer to 24- or 48-hour post-infection treatment (*e.g.*; see Table S5). These times were chosen because they reflect the time of most of the measurements in the dataset used to build the model (Table S3) and also because we are comparing the long-time behaviour (I.e., steady state) of the model. Boolean Networks do not possess an explicit notion of physical time.

We compared the literature with the aggregate of the last two iterations to minimize the effect of oscillatory behavior (see Section B.4) and used the method in Section B.5 to decide if a not was “up” or “not up.”. We computed F1-scores (See Eq. 1) for each of our scenarios.) Table S5 shows the experimental data from literature (columns 4 and 5), and the prediction made by the four scenarios for that measurement (Columns 6-9). Each quantity measured (Column 4) has an equivalent in the model (e.g., cleaved caspase-9 (literature) = CASP-9 (model); p-IRF3 (literature) = IRF3 (model); etc.). The F1-score (See Eq. 1) for the four scenarios is computed directly from the data in Table S5. To assess the likelihood that those F1-scores (See Eq. 1) could be generated by chance, we generated 30 random Boolean networks for each scenario by randomly shuffling the model’s connections (as in Figure S1). We computed the F1 score in these 120 random Boolean networks and counted the proportion of them that had an equal or higher F1 score than the unshuffled ones. We found that random networks from different scenarios had the same average, standard deviation, and distributions (not shown). Therefore, we merged them and computed a single mean and standard deviation. To obtain a more robust estimate, we did not use the empirical data directly, but instead, we used it estimate the mean μ and variance $\sigma ^2$. We then computed the probability that a random model would achieve an F1-score greater than or equal to that of the unshuffled model as the upper-tail probability of the corresponding normal distribution $\mathcal {N}(\mu , \sigma ^2)$. The empirical distribution had strong agreement with a normal curve (not shown).

1 $$\begin{aligned} F1 = \frac{2*TP}{2*TP + FP + FN} \end{aligned}$$

Where TP are the true positives (the sum of factors that were up in the literature and up in the model), FP is the false positive rate (not-up in the literature up in the model), and FN is the false negative rate (up in the literature not-up in the model).

## Results

### Evaluating the Model

**Table 1:** F1-score of the model over several literature datasets not used to build the model. Column 1, scenario and scenario description. Column 2, F1-Score in the whole dataset. Column 3, F1-Score in the “live" COVID dataset. Column 4, F1-score in the viral protein dataset. Column 5, F1 Score in the non-COVID dataset. This dataset evaluates the model’s performance on pathways activated during infection. All computations in this table were done with the data from Table S5

Scenario	F1-Score			
	All	SARS-CoV-2	Viral protein	Non-COVID
1 - increases replication	0.763	0.917	0.769	0.667
2 - increases replication AND inhibits IFN	0.763	0.917	0.769	0.667
3 - inhibits IFN	0.747	0.870	0.769	0.667
4 - None	0.712	0.762	0.769	0.667

**Table 2:** The p-values of the different scenarios shown in Figure 1. The p-value was computed as the percentage of random models that performed equal to or better than the model. The adjusted p-value was computed with FDR using the four p-values from the four scenarios in column 2. Abbreviated descriptions in the table are “increase replication" = “mTORC1 increase viral replication" and “inhibits IFN" = “mTORC1 inhibits type I IFN expression".

Scenario - mTORC1 action	p-value	adjusted p-value
1 - increases replication	0.0026	0.0049
2 - increases replication AND inhibits IFN	0.0026	0.0049
3 - inhibits IFN	0.0037	0.0049
4 - None	0.0076	0.0076

To evaluated the model, we selected a set of papers not used to build the model (See Table S5). The evaluation dataset with the data from these papers can be divided into three groups: “live" SARS-CoV-2 data, viral protein data (*i.e.*, cells incubated with N or S protein), and others. The last one is comprised of cells not incubated with virus or viral proteins but receiving other stimuli that evaluate pathways relevant for the infection.

These three sub-datasets measure outcomes, such as p-STAT1, p-AKT, and IFN protein expression, in response to various stimuli, including infection with live SARS-CoV-2 (“live" SARS-CoV-2 dataset), incubation with viral protein (viral protein dataset), and other stimuli, such as hypoxia, TNF, and Angiotensin-II. Table 1 shows the F1 score for each scenario on this independent dataset. To assess the odds that such F1 scores could be generated by chance, we created a set of random models by shuffling the edges (Figure S1) and computed the likelihood that a random model would achieve an F1-score equal to or higher, than the non-shuffled four scenarios, over the whole dataset (Table 2). The random models had an average F1-score of 0.408 and a standard deviation of 0.139.

We found that scenario 1-3 had a good F1-score in the live SARS-CoV-2 dataset (>0.87), while in the viral protein dataset, all four scenarios had an F1 score of 0.769. In the non-COVID dataset, they showed low performance (0.667). The non-COVID dataset queries pathways involved in SARS-CoV-2 responses, but the model was not explicitly designed for this context. The F1 scores of the four scenarios over the whole dataset were better than 99.2-99.5% of the random models (Table 2), even when adjusting for multiple tests. These results show that this model can reasonably predict outcomes related to the pathways critical to this study.

### In-house empirical data: the Virus/mTORC1/IFN pathway

**Fig. 3:**
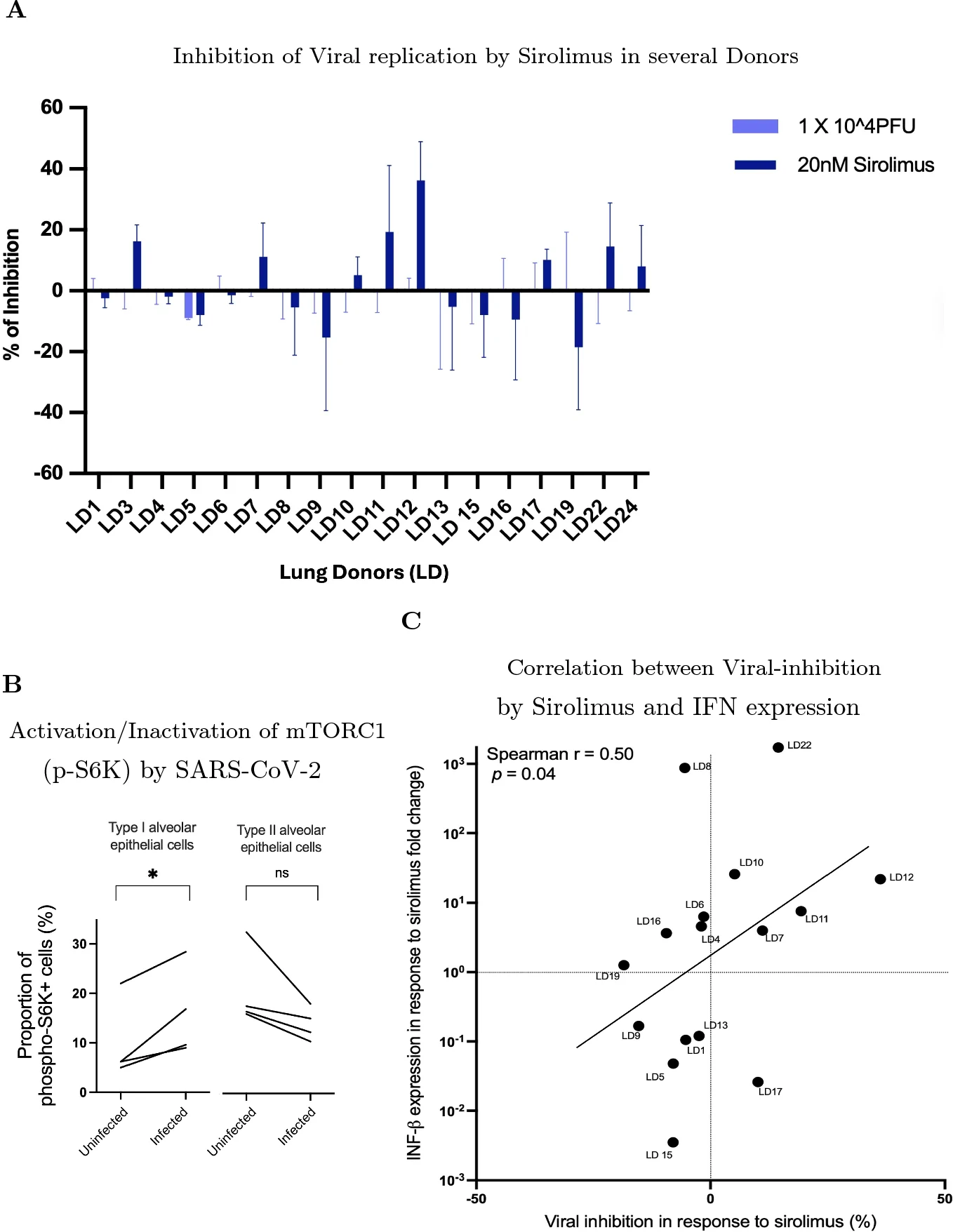
Tissue culture infected with SARS-CoV-2 (in-house data). A: inhibition of SARS-CoV-2 replication in human lung culture of several donors infected with SARS-CoV-2. Each bar represents a donor, which is the average of a triplicate (technical replicate). The difference of the means was assessed with a t-test. B: comparison of p-S6K (mTORC1 reporter gene) activation between control and SARS-CoV-2-infected type-I and II pneumocytes. Each line represents an independent biological replicate from a different donor. For each donor, experiments were performed in triplicates (technical replicate), and values shown are the mean. The difference of the means was assessed with a t-test. C: Correlation between viral inhibition and type-I IFN expression in the human lung culture infected with SARS-CoV-2. Each point is a different donor (biological replicate) and represents the average of a triplicate (technical replicate). Correlation calculated by Spearman test. * p<0.05 (color figure online)

Figure 3 presents experimental evidence from ex vivo human lung tissue cultures. First, SARS-CoV-2 infection induces the activation of mTORC1 in type I pneumocytes, as shown by increased p-S6 staining (Fig. 3A). Second, treatment with Sirolimus, an mTORC1 inhibitor, leads to a reduction in viral replication in tissues from a subset of donors (Fig. 3B). Third, across these samples, the degree of viral inhibition by Sirolimus correlates positively with type I IFN expression (Fig. 3C). Together, these results support a mechanistic hypothesis wherein SARS-CoV-2 activates mTORC1, which in turn promotes viral replication, and it also suppresses IFN expression.

**Fig. 4:**
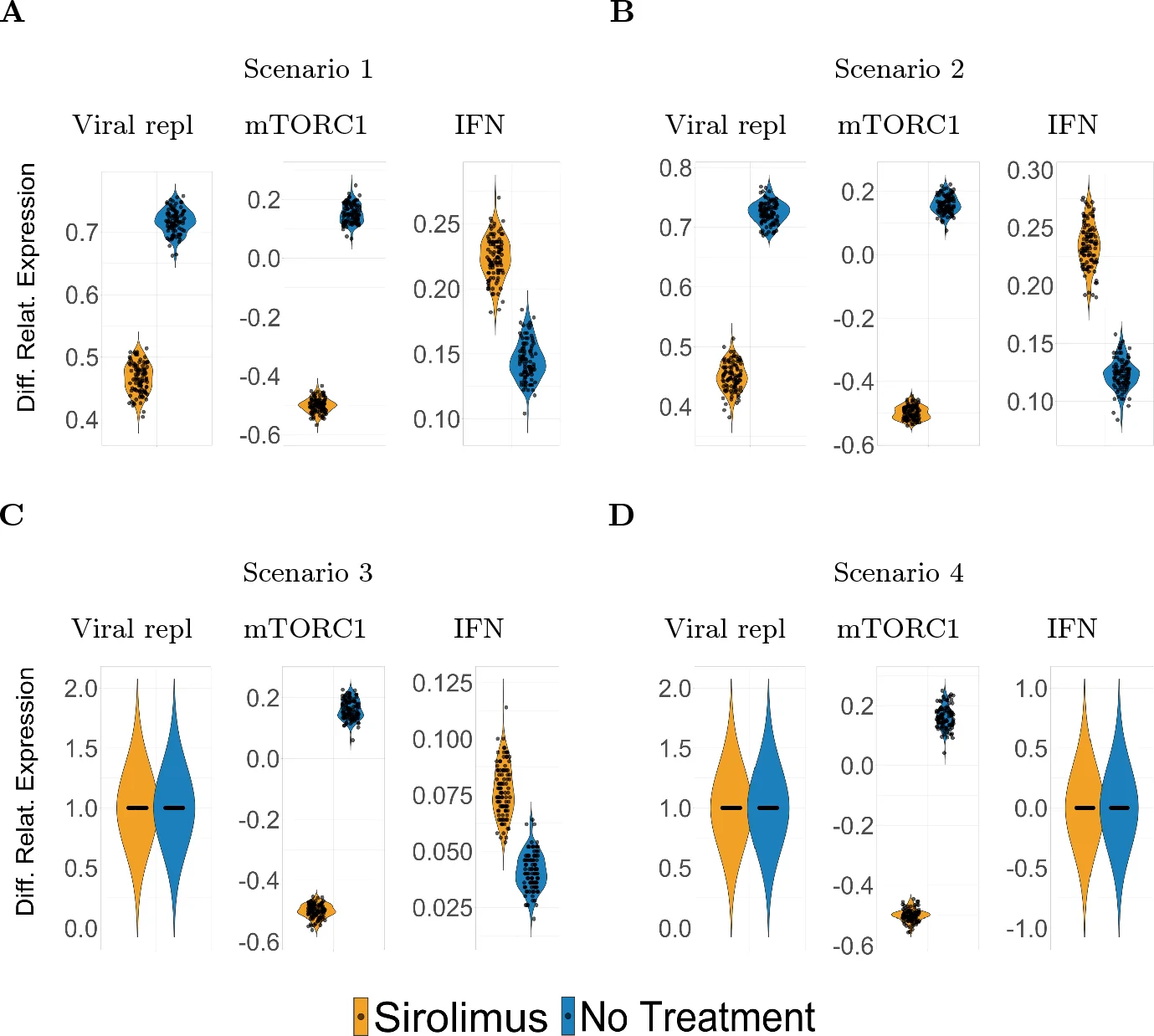
Violin plots showing that viral replication with Sirolimus lower than without and IFN higher in the model. The plots compare the relative activation/expression of simulations of cells infected with SARS-CoV-2 (blue) and cells infected with SARS-CoV-2 and treated with Sirolimus (gold). The y-axis measures the difference in expression between the infected cells and the control (uninfected). A negative number represents decreased activation, and a positive one increased activation. The graphs show predictions for viral replication, mTORC1 activation, and type-I IFN expression. All the comparisons are statistically significant with $p < 10^{-60}$, except for viral replication in scenarios 3 and 4 and IFN in scenario 4 (p>0.3). All comparisons show differences of more than 50% except for viral replication in scenarios 3 and 4 and IFN in scenario 4 (0%). Each experiment contains 100 points - 100 independent and identical ensembles of 500 networks. Scenario 1: mTORC1 increases viral replication; Scenario 2: mTORC1 increases viral replication AND inhibits type-I IFN expression; Scenario 3: mTORC1 inhibits type-I IFN expression; Scenario 4: mTORC1 has no direct effect on viral replication or type-I IFN expression (Color figure online)

To evaluate whether our computational model could recapitulate these experimental findings, we tested the four mechanistic scenarios described earlier. Specifically, we queried whether the model could qualitatively reproduce the behavior observed in Fig. 3C: conditions representing virus + Sirolimus treatment should result in reduced viral replication and increased IFN levels relative to virus alone. Simulation results in Fig. 4 show that Scenarios 1 and 2 capture this qualitative behavior. In Figs. 4A-B, viral replication is lower with Sirolimus than without, while IFN is higher.

Although Scenario 2 directly implements the proposed hypothesis (mTORC1 promotes viral replication and inhibits IFN – M1 and M2), Scenario 1 does not. This scenario implements direct mTORC1 induction of viral replication, but no direct IFN inhibition (M2 only). However, we can still not discard scenario 3 (Fig. 4C). In that scenario, viral replication between Sirolimus-treated and untreated was the same; nevertheless, viral replication showed no variance, which suggests that this could be a model artifact. To test that hypothesis, we introduced a stochastic shifting of node values during the simulations. At each iteration, each node in the Network had a 15% chance of randomly shifting its value.With this new approach, scenario 3 reproduced the empirical data while results remained unchanged for scenarios 1 and 2 (Figure S3). Scenario 3 implements direct mTORC1 inhibition to IFN, but no direct viral replication induction (M1 only). On the other hand, scenario 4 (mTORC1 does not directly induce viral replication nor inhibit IFN expression) was not able to reproduce the data in Fig. 3, neither with or without the random node shifting (Fig. 4D and S3D). This shows that the original hypothesis (both mechanisms) is not necessary to explain the data. Instead, is more plausible that mTORC1 increases viral-replication and increased viral replication inhibits IFN signaling in several points.

A potential caveat in our model is the known distinction between free IKK subunits (IKK-α and IKK-β) and the IKK complex (IKK-$\alpha /\beta /\gamma$): the former activate mTORC1/2, while the latter are primarily responsible for NF-κB signaling. To test whether this distinction mattered, we constructed a model variant in which we removed the IKK→NF-κB and IKK→AKT connection and substituted them with direct TLR→NF-κB and TLR→AKT. At the same time, we also removed TLR→IKK and AKT→IKK. The only remaining IKK activator is TNF. This is in accordance with the signal flow from TNF to IKK-β to mTORC1/2 shown in KEGG (Kanehisa Laboratories yy). The direct connections from TLR to NF-κB and AKT simulate a flow of signal through an IKK-complex that is not explicitly represented. These changes did not alter the results (Figure S4), showing the robustness of the model under minor variation in modeling choices.

**Table 3:** Percentage of model variants that still reproduce the data in Figure 3 (*i.e.*, viral replication with Sirolimus lower than without and IFN higher, at a $p < 10^{-5}$). Column 1: scenario and a brief description. Column 2: percentage of variants (out of 6328) that reproduce the empirical data. If the binomial distribution is assumed, all proportions are at least eight sigmas apart.

Scenario - mTORC1 action	% of variants
1 - increases replication	81.3%
2 - increases replication AND inhibits IFN	97.5%
3 - inhibits IFN	75.0%
4 - None	0.0%

### Network edge perturbation analysis

While Figure S4 tested the model’s robustness to a set of particular changes, we next assessed its robustness to a wide range of perturbations. These perturbations may reflect modeling uncertainty and also biological variability. For example, Fig. 3 shows variability among donors concerning viral inhibition through Sirolimus treatment, IFN expression, and mTORC1 activation.

We did a systematic set of perturbations in the model, consisting of either adding (knock-in) or removing (knock-out) edges. This procedure resulted in the creation of 6,328 variant networks for each scenario (*i.e.*, 25,312 total variants), as explained in Section B.6. Table 3 shows the frequency with which each variant in each scenario qualitatively reproduced the result in Fig. 3. We considered that a variant reproduces the result in Fig. 3 if viral replication with Sirolimus was smaller than viral replication without Sirolimus, and IFN expression with Sirolimus was higher than without. To assess the differences, we used a t-test with $p < 10^{-5}$.

**Fig. 5:**
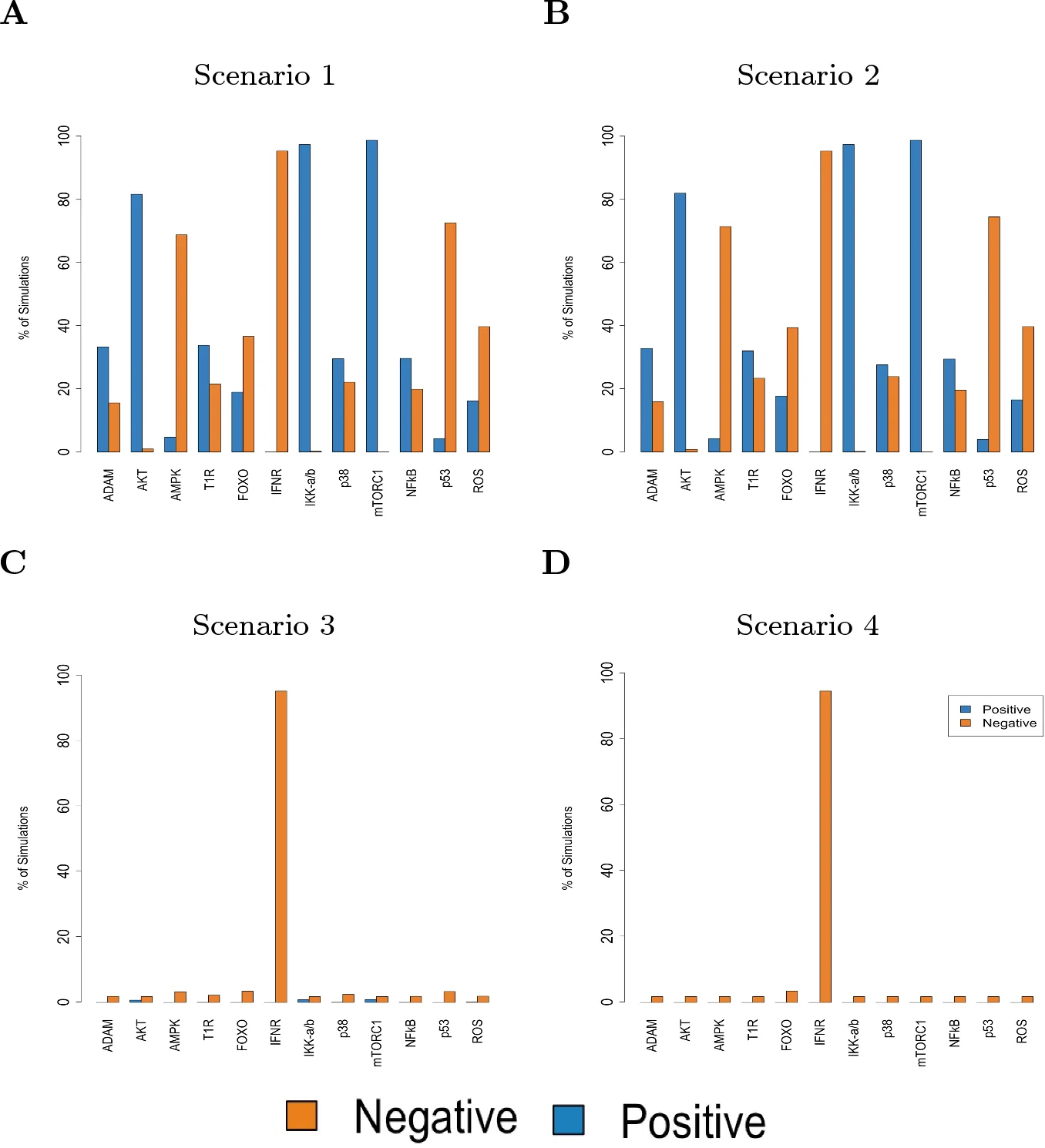
Percentage of simulations that lead to a 5% increase (blue) or decrease (orange) in the viral replication rate (measured here by the frequency of networks in the ensemble that had Viral_repl activated) of upon node activation (x-axis). Each of the 25,312 variants was subjected to the constant activation of the twelve nodes of interest. Scenario 1: mTORC1 increases viral replication; Scenario 2: mTORC1 increases viral replication AND inhibits type-I IFN expression; Scenario 3: mTORC1 inhibits type-I IFN expression; Scenario 4: mTORC1 has no direct effect on viral replication nor type-I IFN expression (Color figure online)

Scenarios 1, 2, and 3 are highly robust to small structural changes, with more than 75% of the cases still reproducing the results from Fig. 3 (Table 3). Scenario 2 (mTORC1 induces viral replication and inhibits IFN expression – M1 and M2) is the more robust (97.5%). This is expected since this scenario has a backup mechanism for reproducing that phenotype. Scenario 1 (mTORC1 induces viral replication but does not directly inhibit IFN expression – M2 only) is the second most robust (81.3%). This suggests that scenario 1 is a stronger candidate than scenario 3 (75%). Not surprisingly, none of the scenario 4 variants reproduced the results from Fig. 3. Even if we use a much looser alpha of 0.05 (instead of $10^{-5}$), only 0.126% (not shown) of the variants would meet the criterion. This indicates that the lack of any mechanism such as M1 or M2 is insufficient to meet the criterion.

The perturbation analysis described in Section B.6 was also used to assess how central nodes in the model affect viral replication. We select the twelve nodes seen in Fig. 5 based on their centrality and pathway representativeness (see Section B.6.2). In this analysis, we measured the number of times knocking in those nodes resulted in an increase or decrease in viral replication of more than 5%. The 5% threshold was a compromise between biological significance and sensitivity.

As seen in Fig. 5, when analyzing the model under a wide range of perturbations, mTORC1 knock-in led to a 5% or more increase in viral replication (measured here by the frequency of networks in the ensemble that had Viral_repl activated) in most scenario 1 and 2 variants (>98%), almost none in scenario 3 (0.9%), and none in scenario 4. Likewise, factors upstream of mTORC1, such as AKT, AMPK, IKK, and p53, were present in scenarios 1 and 2 (68-97%) but not in 3 and 4, where IFN was dominant (88-95%). The negative result in scenario 3 of Fig. 5 is another corollary of the lack of variability in scenario 3.

Figure S8 expands the result of Fig. 5 to other outputs of interest, inflammation measured by IL6, IFN expression, and apoptosis. The results are not segregated by scenario but are instead the aggregate of all four (*i.e.*, all 25,312 variants). Most of the tested factors had an influence on IL6 (>20%). With IKK, NF-κB, p38, T1R, and ROS being the top activators (>80%) and FOXO3A and p53 the top inhibitors (>80%). Likewise, many factors influenced apoptosis. With T1R (renin/angiotensin), FOXO3A, IFN, and p53 being the main inducers (>75%). And NF-κ B and IKK are the main inhibitors (>55%). Notably, the analysis revealed a multifaceted role for FOXO3A: it exhibited moderate anti-viral effects (20% of variants, likely mediated via IFN), although in 9% of the variants it had the opposite effect. Furthermore, FOXO3A was the strongest inducer of IFN expression (≈69%), the most consistent inhibitor of IL-6 (≈97%), and also exhibited strong pro-apoptotic activity (≈79%). These results position FOXO3A as a key immunoregulatory hub within the model, with coordinated influence over anti-viral, inflammatory, and apoptotic pathways.

### Model Adjustment

Next, we tested whether adjusting the model to the evaluation data would change the main conclusion in this paper. While the model performed well overall (better than 99% of random model variants on the whole data set) and very well on the “live" COVID data (F1-Score of 087-0.91 Scenarios 1-3), it did not perform very well on the non-COVID data (F1-Score 0.667). We adjusted the model to the evaluation dataset by a process of non-blind literature search (Table S3). To be more specific, we returned to the literature and searched for new connections that would improve the model’s F1-score on the evaluation dataset. Therefore, the adjustment process was a manual process similar to the model-building one, with the caveat that it was not blind and the quantity being optimized was the F1-score (see Table S1). We tested the adjusted model, which would yield substantially different results, and found that it cast the same predictions concerning the empirical data (Fig. 3). Therefore, this result again demonstrates the robustness of the model’s predictions.

### Model Expansion and Model Reduction

To address potential bias in the model, we adopted two opposing strategies. First, we expanded the model with interactions from the COVID-19 Disease Map (Table S6). This expanded model included important new connections that could change the model’s dynamics, such as viral inhibition of NFkB and MAPK, and the ability of those pathways to induce an IFN response. While we did not test the overall dynamics of the model, Figure S6 shows that this expanded model produced the same results as the other variants (Figs. 4, S3, S4, S5). We also tested this new model against our evaluation dataset, and it had the same score (not shown). Opposite to that, we tried a simple procedure of model reduction. Figure 1 already shows a condensed version of the model. The only modifications we made to the model presented in Fig. 1 were the expansion of the IFN pathway, necessary to support the dual role of viral replication (activatory and inhibitory - see Table S4), and the ablation of the Apoptosis pathway. Since the goal here was only to test if the reduced model reproduces the results in Fig. 4, and Apoptosis does not influence other pathways (see Fig. 1), it was not necessary to code it. Table S4 shows the equations for this reduced model, and Figure S7 shows that this model also qualitatively produced the same results as the others.

## Discussion

In this study, we developed a mechanistic model of the intracellular response to SARS-CoV-2 in pneumocytes. The model integrates in-house experimental data from ex vivo tissue cultures and curated literature describing signaling pathways relevant to viral infection, inflammation, metabolism, and immune regulation. Importantly, the model was evaluated against an independent literature dataset not used in its construction. Part of the evaluation process included the construction of Random Networks using random perturbations of model structure (i.e., edges shuffling - Figure S1), resulting in models of comparable topology and complexity. The process of model evaluation used here is similar to that employed in several existing studies (*e.g.*, Palma et al. (2018); Pedicini et al. (2010); Marku et al. (2020)), with the added step that we measured the likelihood that our result could have been generated by chance. We found that our four scenarios performed better than 99% of such Random Networks.

Our in-house experimental data revealed that mTORC1 activation correlates positively with viral replication and negatively with IFN-β expression (Fig. 3). This led to the hypothesis that mTORC1 both promotes viral particle synthesis and suppresses type-I IFN expression. While the role of mTORC1 in promoting viral replication is supported by mounting evidence in both patient samples and *in vitro* models (Fattahi et al. 2022; Basile et al. 2022), its involvement in regulating IFN remains less well established. To our knowledge, this is the first report of a negative correlation between mTORC1 and IFN-β in the context of SARS-CoV-2 infection. The literature presents mixed findings for other viral systems. For instance, Alain et al. (2010) reported that mTORC1 enhances IFN responses against oncolytic viruses, whereas (Liu et al. 2017) found that inhibiting mTORC1 increased type-I IFN production in macrophages infected with PRRS virus. Similarly, Fattahi et al. (2022) proposed that mTORC1 contributes to cytokine overproduction, suggesting a broader role in inflammatory responses. Meanwhile, the ability of SARS-CoV-2 proteins to suppress type-I IFN signaling is well documented (Salman et al. 2021; Tolomeo et al. 2022).

To evaluate alternative mechanistic explanations for our experimental observations, we constructed four distinct model scenarios, each encoding a different hypothesis:

1. Scenario 1: mTORC1 enhances viral replication (Basile et al. 2022; Fattahi et al. 2022).
2. Scenario 2: mTORC1 enhances viral replication and inhibits IFN expression (our working hypothesis).
3. Scenario 3: mTORC1 inhibits IFN expression only (Fattahi et al. 2022).
4. Scenario 4: mTORC1 has no direct effect on viral replication nor type-I IFN expression.

The simulation results challenged the original hypothesis (Scenario 2). While the results from this scenario are consistent with experimental data presented in this paper, the results from Fig. 4A show that the presence of both mechanisms (mTORC1 enhances viral replication and inhibits IFN expression) is not necessary to explain the phenomenon. Instead, the enhancement of viral replication by mTORC1 alone is sufficient to both decrease viral load and increase IFN expression upon sirolimus treatment. The model reveals that, if mTORC1 increases viral replication, and viral replication inhibits IFN expression, then mTORC1 can inhibit IFN by proxy. One can observe this from the interactions in Fig. 2; however, it is not entirely clear from inspecting Fig. 2 that this is what happens, since viral replication both inhibits IFN expression (Zhang et al. 2022; Salman et al. 2021; Sodeifian et al. 2022) and triggers it via RIG-I (Yamada et al. 2021). Therefore, we need the simulations shown in Figure 4, S3, S4, S5, S6, and S7, to confirm that this paradoxical relationship between the virus and IFN leads to mTORC1 inhibiting IFN by proxy. This model favors scenario 1 over the others because it is based only on interactions that were already observed for other viral infections (see Fattahi et al. (2022); Basile et al. (2022)), while scenarios 2 and three require positing the existence of mTORC1 as an inhibitor of IFN, which is not fully supported by the literature (see Alain et al. (2010); Liu et al. (2017)).

To address whether small changes in the model could substantially change the results, we considered several model variants. Those variations were created on top of the four scenarios already discussed. While the four scenarios were an attempt to find the best explanation for the empirical data, this new set of variations was an attempt to gauge the robustness of the results in Fig. 4. We can divide the sources of variations into three. First, a reimplementation of the IKK signaling to account for the uncertainty in the mechanism of action of this kinase. Second, we fit the model to the evaluation data. Third, we did a systematic set of edge knockouts and knock-ins. We found that the first two did not change the results (Figures S4 and S5), while the latter reproduced the results in most of the variants (81.3% scenario 1 and 75% scenario 3). This data also reinforces scenario 1 as the best explanation by showing that it is more robust than scenario 3.

Subsequently, we tested the effect of an array of 12 factors in viral replication and found that, to a lesser or greater extent, all twelve impacted the infection in scenarios 1 and 2. With mTORC1, type I IFN, IKK-$\alpha /\beta$, FOXO3A, and AKT being the most prominent, affecting more than 75% of variants each. The literature has predominantly focused on mTORC1, AMPK, and PI3K/AKT as candidate drug targets in SARS-CoV-2 infection (Basile et al. 2022; Fattahi et al. 2022), but our analysis identifies IKK-$\alpha /\beta$ and FOXO3A as potentially important as well. The latter one, although it only reduced the viral replication in approximately 40% of the variants in scenarios 1-2, had strong effects on inflammation and apoptosis (>75% of variants across all scenarios).

IKK-$\alpha /\beta$, traditionally viewed as an activator of NF-κB, was found in our simulations to consistently promote mTORC1 activity and suppress IFN expression. This suggests that inflammation may facilitate viral replication, possibly through mTORC1 activation. IKK has previously been proposed as a target for COVID-19 therapy due to its role in inhibiting apoptosis via NF-κB (Fagone et al. 2020), our analysis also finds a strong relation between IKK and apoptosis (Fig. S8). This kinase is a suppressor of IFN expression and a trigger of viral replication (Figures S8 and 5). While IKK is an inhibitor of FOXO3A, a pro-IFN transcription factor, its effect on viral replication was strongly attenuated in scenarios 3 and 4 ($\approx 1.8\%$ of variants of scenarios 3-4 compared to 97% in scenarios 1-2). This suggests that the main action of IKK in the model is through mTORC1.

FOXO3A was the most interesting of the two targets suggested by the model. Its activation reduced viral replication ($\approx 20\%$ of variants across all scenarios), strongly induced IFN expression (>60% of variants), reduced inflammation (>90%), and induced apoptosis in many variants (>75%). It is worth noting that many of these effects are mediated by the hypothesis that FOXO3A induces IFN expression (Gimenes-Junior et al. 2019). All these effects suggested by our model are also suggested by Cheema et al. (2021), which proposes FOXO as a target with potential anti-viral and anti-inflammatory properties. Similarly, Antonellis et al. (2024) shows that SARS-CoV-2 induces AKT, which inactivates FOXO3A. FOXO3A inactivation was shown in their work to promote viral replication, although the mechanism differs from the one in our model. In the results from Antonellis et al. (2024), the viral replication induction happens due to Ca2+ influx upon FOXO3A inactivation, while in our model, it is due to the reduction in IFN expression.

The model was constructed entirely from published literature, an approach that entails both strengths and limitations. On the one hand, literature-driven modeling provides a structured synthesis of existing knowledge and enables explicit testing of current mechanistic scenarios. On the other hand, such models are inherently subject to modeler bias in the selection and representation of interactions. To gauge this bias, we expanded the network to include additional interactions documented in the COVID-19 Disease Map (Table S6) and tested the impact on model predictions. Conversely, we reduced the model to a minimal core consisting only of the major pathways depicted in Fig. 1. While the reduced model sacrifices the specificity of individual molecular interactions, it preserves the higher-level relationships among key pathways. We found that both the expanded and reduced models reproduced the experimental observations. This consistency indicates that the principal findings are robust and unlikely to be a modeling artifact. As a limitation, we point to the approach that is new, and it would be interesting to see if the conclusions found here are reproduced in other modeling paradigms, such as ODEs and PDEs. The average of multiple simulations could also hide different trajectories and future work could dig into that. However, the approach used here is not much different from that used by Palma et al. (2018). From an experimental point of view, the tissue culture is obtained from autopsies and is not from healthy individuals. However, this is not much different from most cell cultures that are immortalized cancer cells.

In summary, our computational model is based on an extensive literature review that captures and explains the experimentally observed relationship between mTORC1 activity, viral replication, and IFN suppression in SARS-CoV-2-infected pneumocytes. While it shows that either or both proposed mechanisms can explain the empirical data, our model strongly suggests that direct mTORC1 induction of viral replication and not direct inhibition of type I IFN is the correct one, because this is the most parsimonious.

## Supplementary Information

Below is the link to the electronic supplementary material.

Supplementary file 1 (pdf 8490 KB)

## Declarations

### Code availability

The model was implemented in Java, and the code can be found at https://github.com/deassisinfo/PneumocytesNet.git.
