# Supplementary material for: mTORC1 Promotes SARS-CoV-2 Replication and Attenuates Type I Interferon Responses in the Alveolar Epithelium: A Computational and Experimental Study
Source: Bull Math Biol. 2026 Sep 2;88(10):171. doi: 10.1007/s11538-026-01701-8 (PMC13538202; doi:10.1007/s11538-026-01701-8)
Supplement: Supplementary file 1 [file 11538_2026_1701_MOESM1_ESM.pdf]

1 Appendix A: Supplementary Figures and Tables

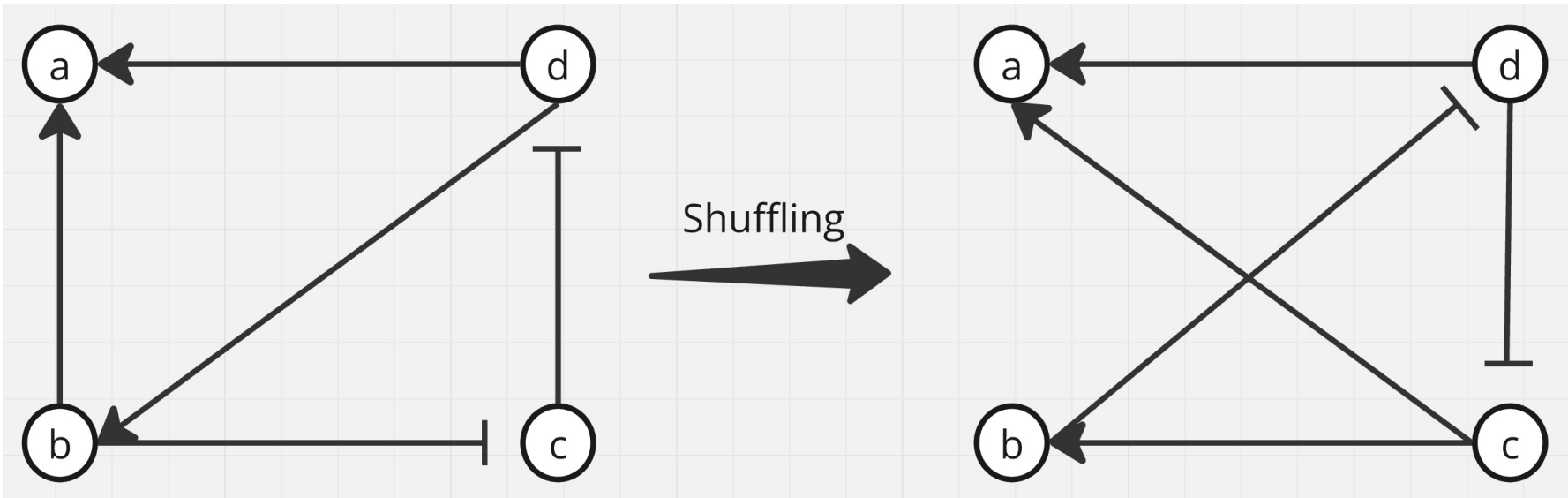

Fig. S1 Figure showing the shuffling process used to create random networks. The shuffling preserves the in-degree of each node as well as the number of in-activators and in-inhibitors.

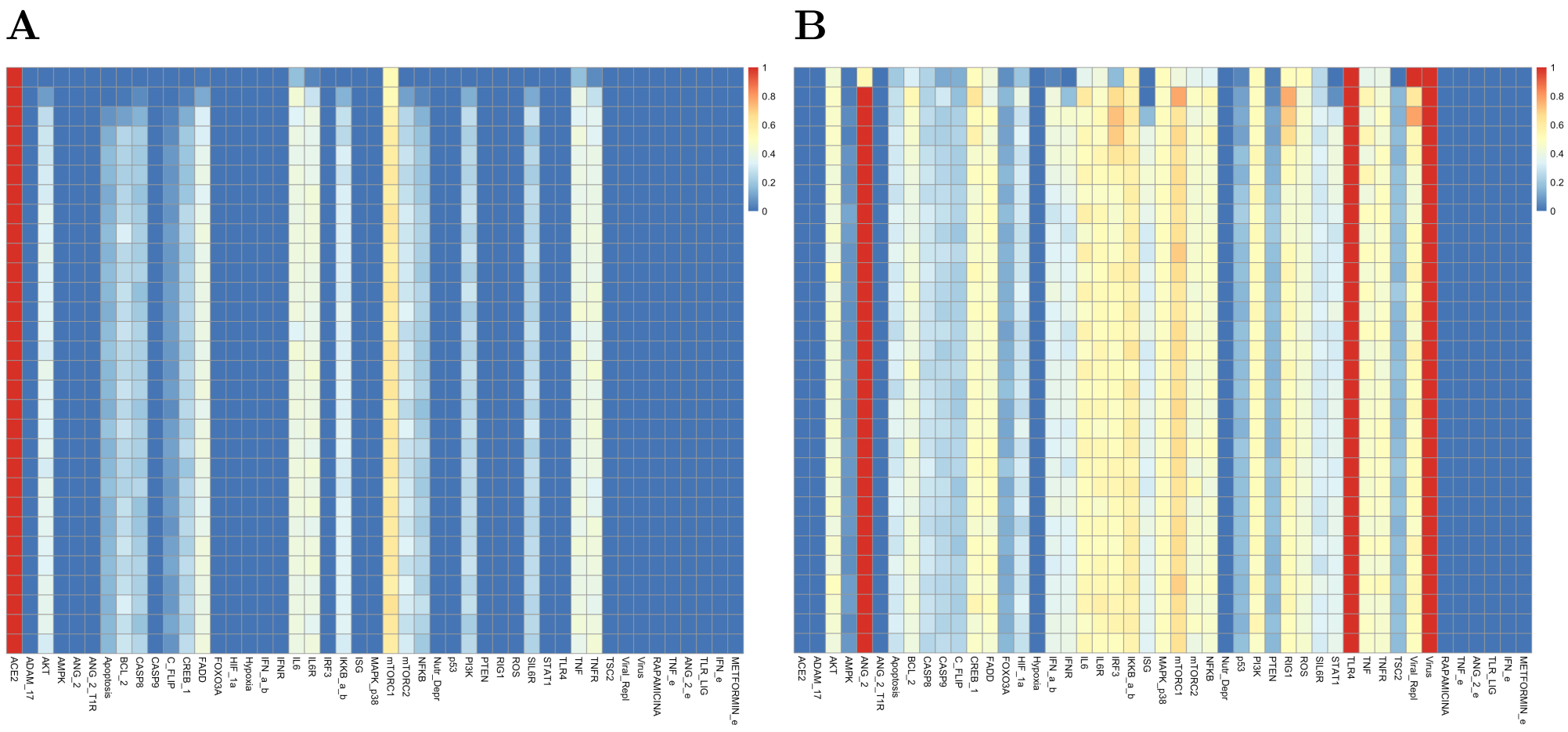

Fig. S2 Heatmap with an sample output of an ensemble of 500 Boolean networks. The y-axis represents iterations (top=1, bottom=30), and the x-axis represents the nodes in the Boolean network. A: The ensemble models were initialized with TSC2=1 and ACE2=1 and run for 30 iterations (resting cells). The First line TSC2 is not equal to one because that was the state of the network before the first iteration was run, and the first line shows the state after the first iteration was run. B: The last iteration (iteration 30) of each model in A (resting cells) was updated with virus = 1 (infection) and run for another 30 iterations. The results in A and B are the average.

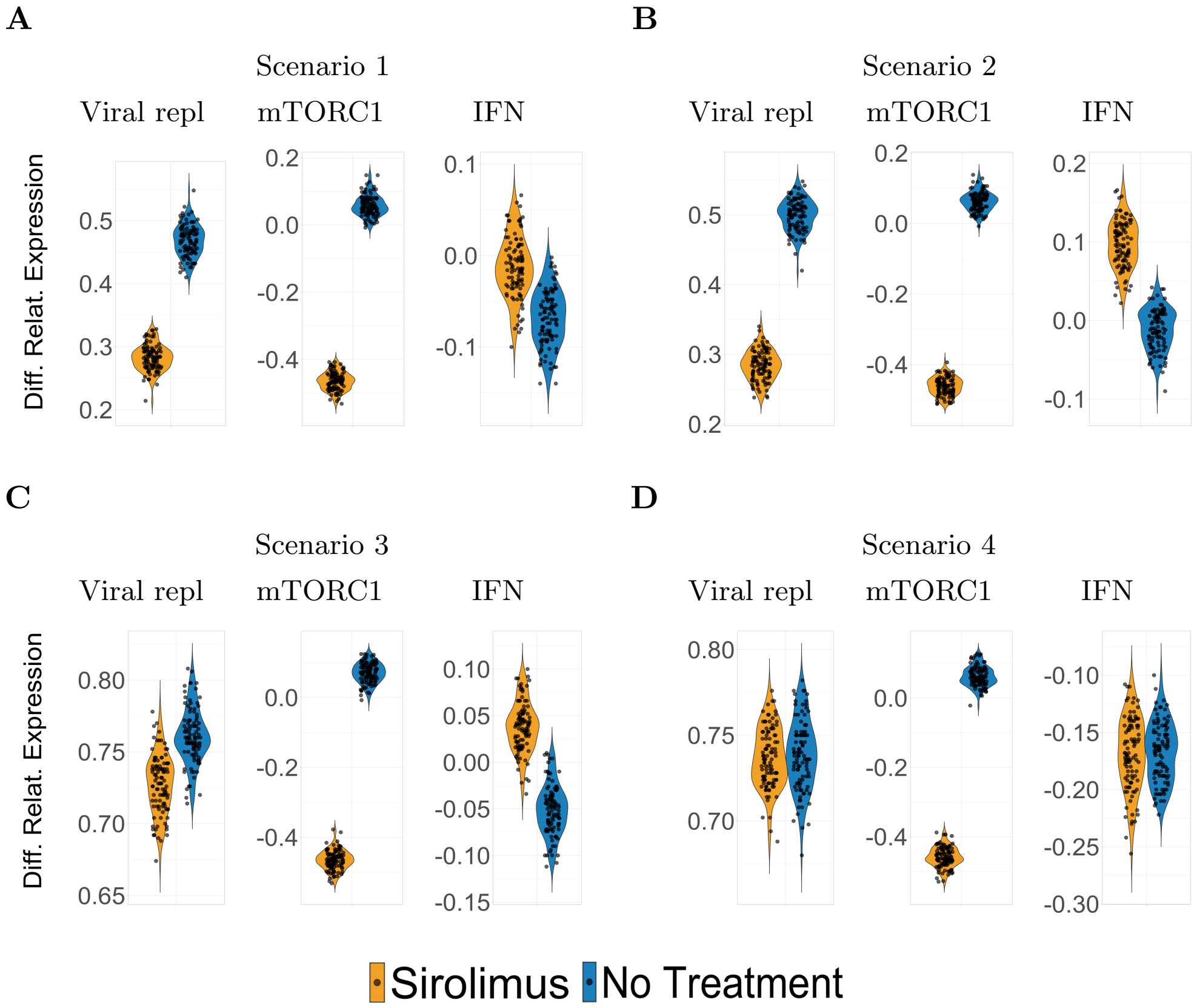

Fig. S3 Violin plots showing that viral replication with Sirolimus lower than without and IFN higher in the model. The plots compare the relative activation/expression of simulations of cells infected with SARS-CoV-2 (blue) and cells infected with SARS-CoV-2 and treated with Sirolimus (gold), using the 15% random shift in node values during the simulation. The y-axis measures the difference in expression between the infected cells and the control (uninfected). A negative number represents lower expression than control, and a positive one higher expression. The graphs show predictions for viral replication, mTORC1 activation, and type-I IFN expression. All the comparisons are statistically significant with $p < 10^{-24}$, except for viral replication in scenarios 4 ($p = 0.557$) and IFN in scenario 4 ($p = 0.703$). All comparisons show differences of more than 50% except for except for viral replication in scenarios 3 and 4 and IFN in scenario 4. Each experiment contains 100 points - 100 independent and identical ensembles of 500 networks. Scenario 1: mTORC1 increases viral replication; Scenario 2: mTORC1 increases viral replication AND inhibits type-I IFN expression; Scenario 3: mTORC1 inhibits type-I IFN expression; Scenario 4: mTORC1 has no direct effect on viral replication nor type-I IFN expression.

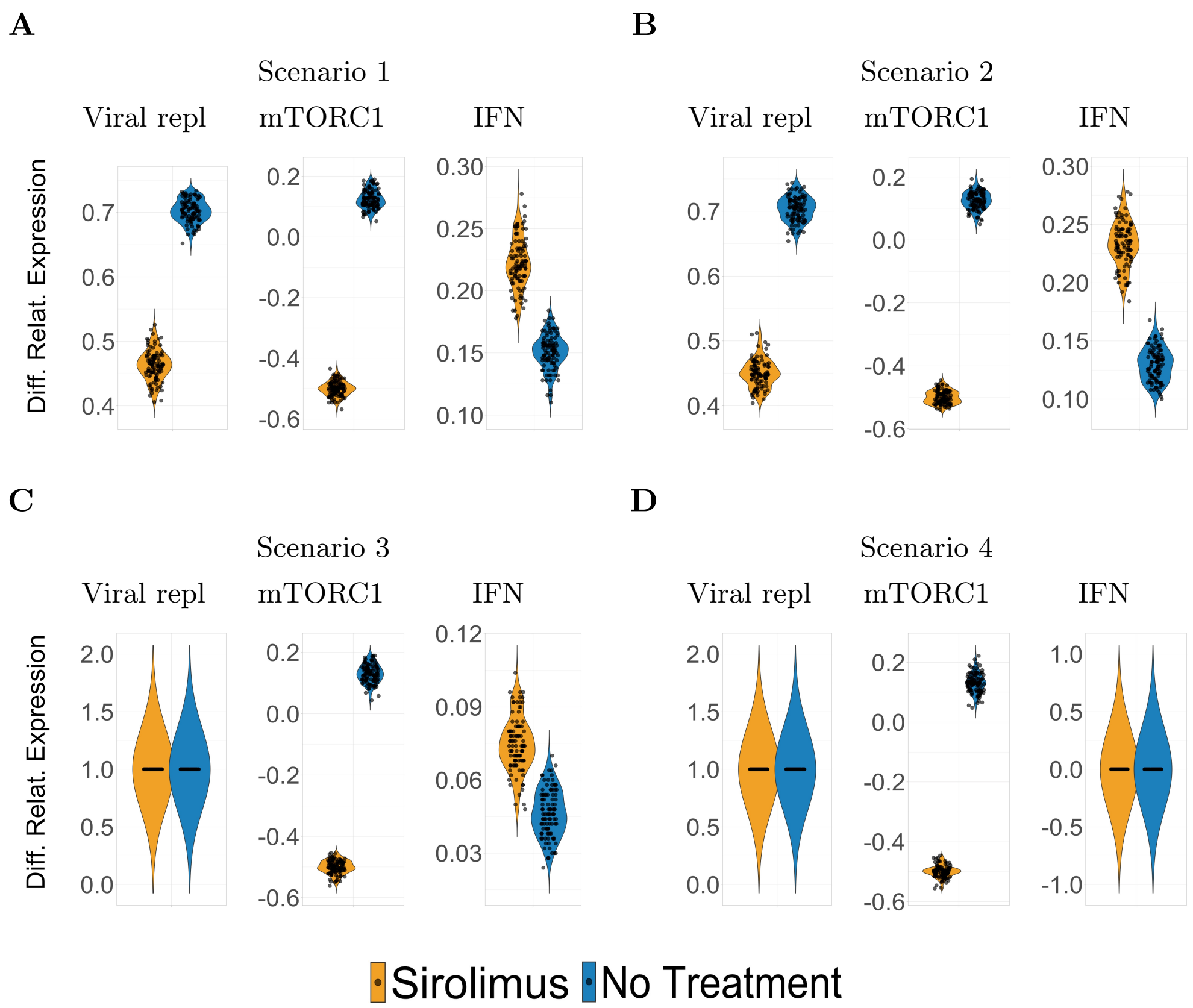

Fig. S4 Violin plots showing that viral replication with Sirolimus lower than without and IFN higher in the model. The plots compare the relative activation/expression of cells infected with SARS-CoV-2 (blue) and cells infected with SARS-CoV-2 and treated with Sirolimus (gold) in our simulations. This plot shows simulations of the model without the connections $IKK \rightarrow$NF-κB, $IKK \rightarrow AKT$, $TLR \rightarrow IKK$, and $AKT \rightarrow IKK$, and with the connections $TLR \rightarrow$NF-κB and $TLR \rightarrow AKT$. The y-axis measures the difference in expression between infected cells and control (uninfected). As before, a negative number indicates lower expression than control, and a positive one higher expression. The graphs show predictions for viral replication, mTORC1 activation, and type-I IFN expression. All the comparisons are statistically significant with $p < 10^{-45}$, except for viral replication in scenarios 3 and 4 and IFN in scenario 4. All comparisons show differences of more than 50% except for except for viral replication and IFN in scenarios 3 and 4. Each experiment contains 100 points - 100 independent and identical ensembles of 500 networks. Scenario 1: mTORC1 increases viral replication; Scenario 2: mTORC1 increases viral replication AND inhibits type-I IFN expression; Scenario 3: mTORC1 inhibits type-I IFN expression; Scenario 4: mTORC1 has no direct effect on viral replication nor type-I IFN expression.

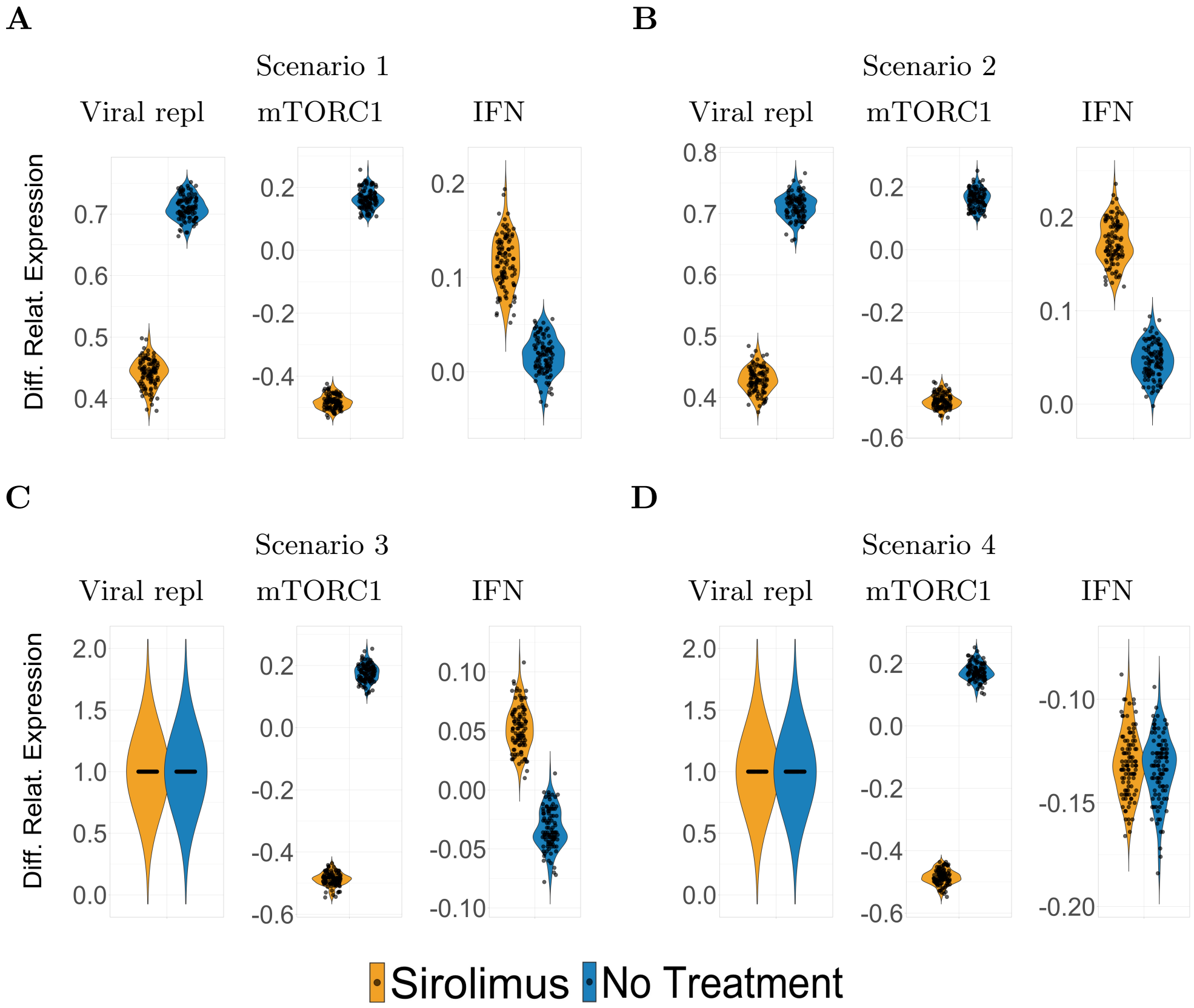

Fig. S5 Violin plots showing that viral replication with Sirolimus lower than without and IFN higher in the model. The plots compare the simulations of relative activation/expression of cells infected with SARS-CoV-2 (blue) and cells infected with SARS-CoV-2 and treated with Sirolimus (gold). Using the adjusted model. The y-axis measures the difference in expression between the infected cells and the control (uninfected). A negative number represents less activity than the control, and a positive one more activity. The graphs show predictions for viral replication, mTORC1 activation, and type-I IFN expression. All the comparisons are statistically significant with $p < 10^{-68}$, except for viral replication in scenarios 3 and 4 and IFN in scenario 4. All comparisons show differences of more than 50% except for except for viral replication in scenarios 3 and 4 and IFN in scenario 4. Each experiment contains 100 points - 100 independent and identical ensembles of 500 networks. Scenario 1: mTORC1 increases viral replication; Scenario 2: mTORC1 increases viral replication AND inhibits type-I IFN expression; Scenario 3: mTORC1 inhibits type-I IFN expression; Scenario 4: mTORC1 has no direct effect on viral replication nor type-I IFN expression.

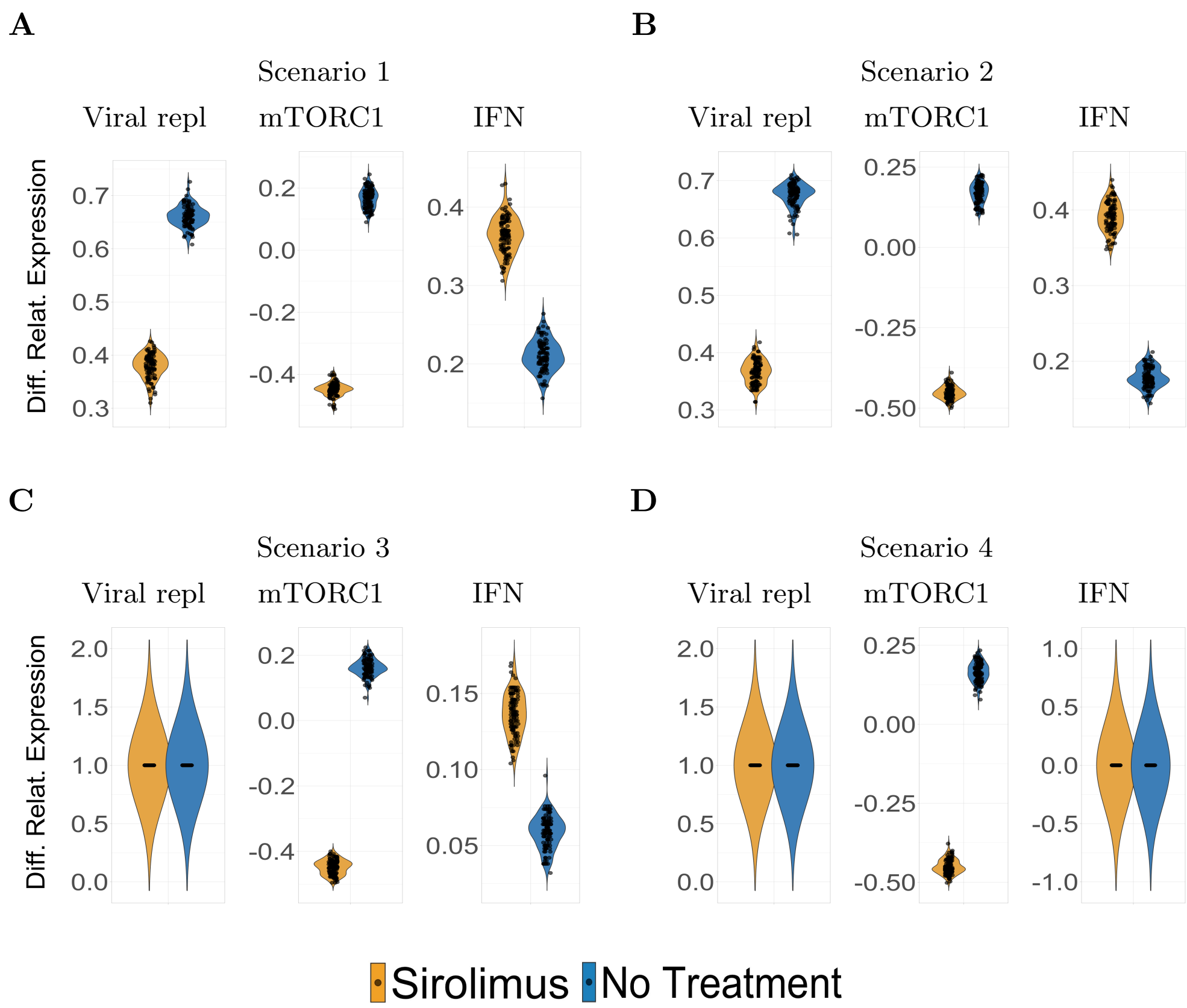

Fig. S6 Violin plots showing that viral replication with Sirolimus lower than without and IFN higher in the model. The plots compare the simulations of relative activation/expression of cells infected with SARS-CoV-2 (blue) and cells infected with SARS-CoV-2 and treated with Sirolimus (gold). Using the model expanded with the pathways shown in Table S6. The y-axis measures the difference in expression between the infected cells and the control (uninfected). A negative number represents less activity than the control, and a positive one more activity. The graphs show predictions for viral replication, mTORC1 activation, and type-I IFN expression. All the comparisons are statistically significant with $p < 10^{-101}$, except for viral replication in scenarios 3 and 4 and IFN in scenario 4. All comparisons show differences of more than 50% except for except for viral replication in scenarios 3 and 4 and IFN in scenario 4. Each experiment contains 100 points - 100 independent and identical ensembles of 500 networks. Scenario 1: mTORC1 increases viral replication; Scenario 2: mTORC1 increases viral replication AND inhibits type-I IFN expression; Scenario 3: mTORC1 inhibits type-I IFN expression; Scenario 4: mTORC1 has no direct effect on viral replication nor type-I IFN expression.

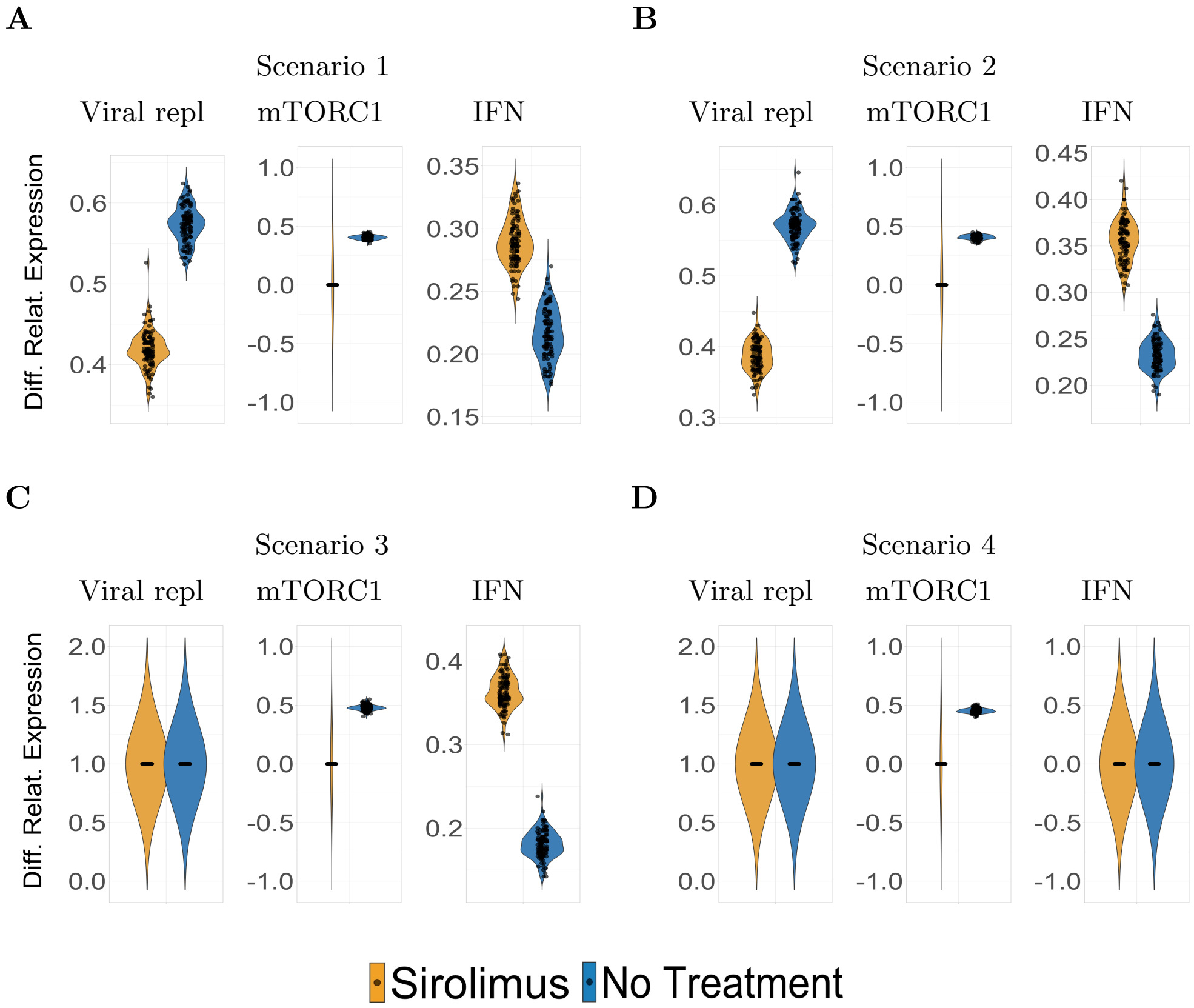

Fig. S7 Violin plots showing that viral replication with Sirolimus lower than without and IFN higher in the model. The plots compare the simulations of relative activation/expression of cells infected with SARS-CoV-2 (blue) and cells infected with SARS-CoV-2 and treated with Sirolimus (gold). Using the Reduced model shown in Figure **??** and Table S4. The y-axis measures the difference in expression between the infected cells and the control (uninfected). A negative number represents less activity than the control, and a positive one more activity. The graphs show predictions for viral replication, mTORC1 (mTOR in this reduced model) activation, and type-I IFN expression. All the comparisons are statistically significant with $p < 10^{-43}$, except for viral replication in scenarios 3 and 4 and IFN in scenario 4. All comparisons show differences of more than 25% except for except for viral replication in scenarios 3 and 4 and IFN in scenario 4. Each experiment contains 100 points - 100 independent and identical ensembles of 500 networks. Scenario 1: mTORC1 (mTOR) increases viral replication; Scenario 2: mTORC1 (mTOR) increases viral replication AND inhibits type-I IFN expression; Scenario 3: mTORC1 (mTOR) inhibits type-I IFN expression; Scenario 4: mTORC1 (mTOR) has no direct effect on viral replication nor type-I IFN expression.

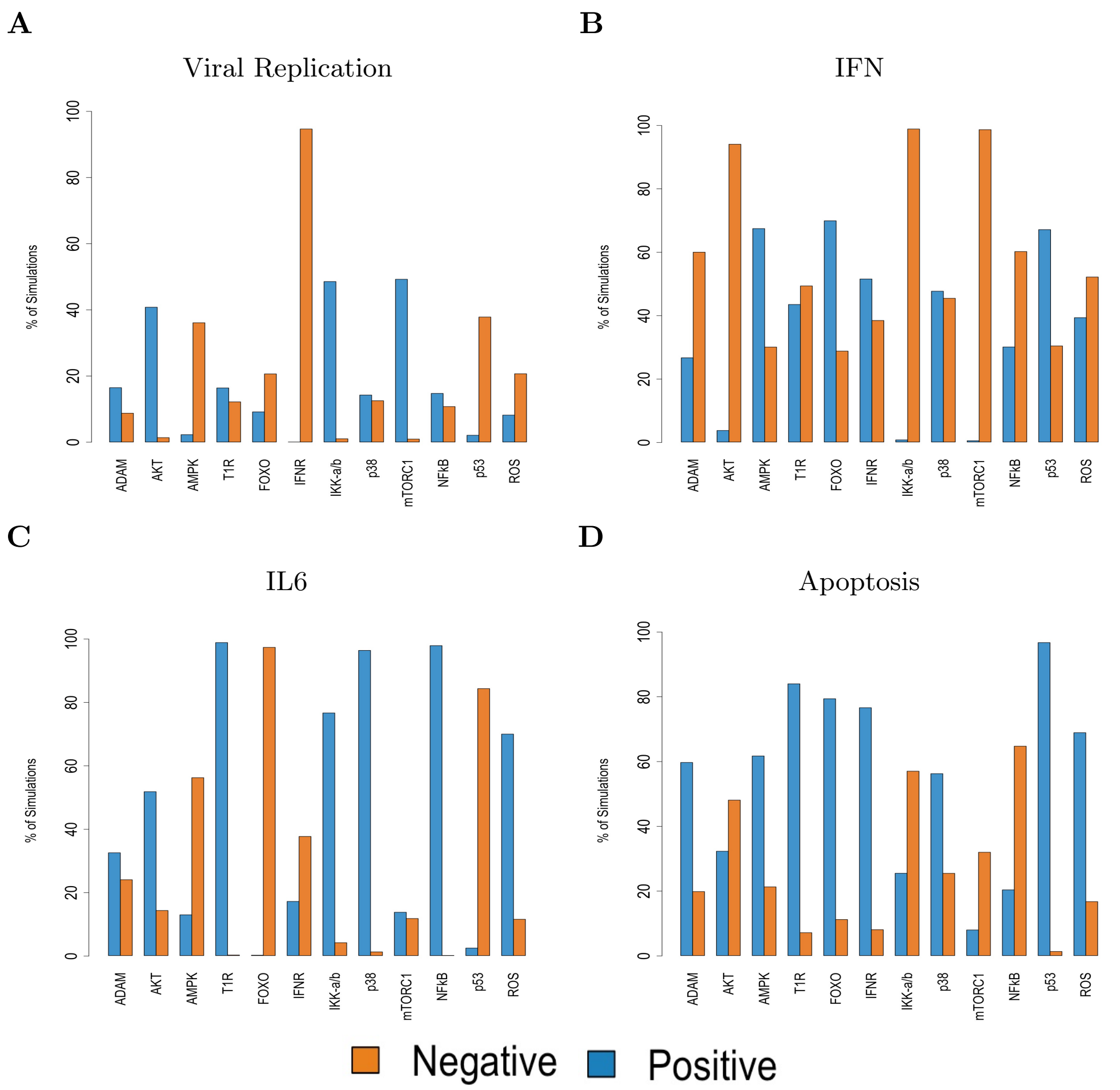

Fig. S8 Percentage of simulations that lead to a 5% increase (blue) or decrease (orange) in the outcomes of interest (measured here by the frequency of networks in the ensemble that had the outcome of interest activated) upon node activation (x-axis) across all four scenarios. A: percentage of simulations that led to a 5% increase or decrease in viral replication. B: percentage of simulations that led to a 5% increase or decrease in type-I IFN. C: percentage of simulations that led to a 5% increase or decrease in IL6. D: percentage of simulations that led to a 5% increase or decrease in Apoptosis

Table S1 F1-score of the adjusted model in several datasets. Column 1, scenario and scenario description. Column 2, F1-Score in the whole dataset. Column 3, F1-Score in the "live" COVID dataset. Column 4, F1-score in the viral protein dataset. Column 5, F1 Score in the non-COVID dataset. This dataset queries pathways that are activated during the infection.

Scenario	F1-Score			
	All	SARS-CoV-2	Viral protein	Non-COVID
1 - increases replication	0.825	0.818	0.923	0.872
2 - increases replication AND inhibits IFN	0.895	0.917	0.923	0.872
3 - inhibits IFN	0.825	0.818	0.923	0.872
4 - None	0.825	0.818	0.923	0.872

Table S2: Target functions in the original model (non-adjusted). Column 1 output node. Column 2 target function. Column 3 description. Column 4 literature support.

Node	target function	Description	Reference
ACE2	$ACE2 - AVG(Virus, ADAM17)$	ACE2 shed upon viral infection, ADAM17 cleaves the ACE2 ectodomain	Grimes and Grimes (2020); Cheema et al. (2021); Mattos Pereira et al. (2024); Mokuda et al. (2020); Zhou et al. (2021)
ADAM17	T1R	Ang II T1R acts on ADAM17	Stepanova (2021); Wang et al. (2022); Mattos Pereira et al. (2024)

AKT	AVG(mTORC2, IKK, PI3K) - PTEN	mTORC2 can phosphorylate AKT, PTEN antagonizes PI3K by dephosphorylating PIP3 to make PIP2 while PI3K and IKK promotes AKT activation	Dan et al. (2016); Carracedo and Pandolfi (2008); Zhao et al. (2017); Basile et al. (2022); Babaev et al. (2016)
AMPK	AVG(Nutr_Depr, p53) - AKT	p53 promote AMPK, and AKT inhibits it.	Hardie et al. (2012); Zhao et al. (2017); Valentine et al. (2014); Hawley et al. (2014); Budanov and Karin (2008)
ANG2	!ACE2	ANG II is cleaved by ACE2 into ANG 1-7	Ni et al. (2020)
T1R	ANG2	Ang II acts on the angiotensin type 1 receptor	Rodriguez-Perez et al. (2021)
Apoptosis	AVG(CASP8, CASP9)	Activation of Casp-8 or 9 result in apoptosis	Porter and Jänicke (1999); Cohen (1997)

BCL2	AVG(NFKB, CREB1) - p53	NF-KB activation results in genes encoding Bcl-2. Increase in p-CREB results in expression of BCL-2, p53 inhibits BCL-2 binding to pro-apoptotic family proteins	Kucharczak et al. (2003); Yang et al. (2023); Wei et al. (2023); Sikder et al. (2014)
CASP8	AVG(FADD, p53) - c-FLIP	FADD recruit and activate Casp-8, p53 promotes cleavage of Procaspase 8, c-FLIP is an inhibitor of Casp-8	Weinlich and Green (2014); Liu et al. (2011); Jyonouchi (1999); Xing et al. (2011)
CASP9	AVG(FOXO3A, p53, ROS) - BCL2	FOXO3A activate Par-4 (BIM) mRNA expression that activates Casp-9, BCL_2 inhibition stimulates BAX/BAK, which promote Casp-9. ROS and p53 promote Casp-9.	Jyonouchi (1999); Xing et al. (2011); Das et al. (2016); Qian et al. (2022); Essafi et al. (2005); García-Cuellar et al. (2020); Lin et al. (2015)

c-FLIP	AVG(NFKB, p53) - AKT	NF-κB activate c-FLIP transcription. p53 leads to c-FLIP and AKT inhibits it.	Lin et al. (2015); Safa (2013); Skurk et al. (2004); Micheau et al. (2001); Bartke et al. (2001); Cha et al. (2010)
CREB1	AVG(AKT, Viral_Repl)	Akt promotes CREB phosphorylation. SARS-CoV-2 activates CREB.	Du and Montminy (1998); Yang et al. (2023)
FADD	TNFR	TNFR1-associated death domain recruits FADD	Chaudhary et al. (1997); Mohd Zawawi et al. (2023)
FOXO3A	AVG(p38, AMPK) - AVG(IKK, AKT)	p38a phosphorylates FOXO3A at Ser-7, AMPK causes FOXO3A up-regulation, IKK phosphorylates/inhibits FOXO3a independent of AKT, FoxO-activated Akt phosphorylates FoxO via feedback inhibition	Marzi et al. (2016); Zhao and Liu (2021); Hu et al. (2004); Dan et al. (2016); Zheng et al. (2014); Basile et al. (2022)
HIF1a	AVG(ROS, Hypoxia, mTORC1)	ROS degrades PHD that promotes free HIF-1a, Hypoxia induces HIF-1a. mTORC1 leads to HIF-1a expression.	Zhu et al. (2021); Ziello et al. (2007)
Hypoxia	Hypoxia	Hypoxia is an external factor	

IFN	AVG(IRF3, FOXO3a) - AVG(mTORC1, Viral_Repl)	IRF 3 binds to Type I IFN Promoters. Foxo3a regulates virus-induced IFN responses in airway epithelial cells.	Salman et al. (2021); Gimenes-Junior et al. (2019); Sodeifian et al. (2022)
IFNR	IFN	INF alpha/beta binds to its receptor	Sodeifian et al. (2022); Mazewski et al. (2020); Salman et al. (2021)
IL6	AVG(NFKB, p38)	NF-κB and p38 promote IL-6 production	Brasier (2010); Klemm et al. (2017); Zarubin and Han (2005); Grimes and Grimes (2020)
IL6R	IL6	IL6 binds to its receptor	Jafarzadeh et al. (2021)
IRF3	RIG1	RIG-1 recognizes viral RNA to activate IRF3	Yamada et al. (2021); Salman et al. (2021)

IKK	AVG(TLR, TNFR, AKT)	TLR4 results in IKKB activation, TNFR activates independently, AKT phosphorylates IKKB	Arora et al. (2019); Huang and Hung (2013); Vollmer et al. (2017); Park et al. (2015); Seo et al. (2014); Dan et al. (2016)
ISG	STAT1 − Viral_Repl	STAT-1 leads to ISG transcription and activation, viral replication can cause ISG dysregulation by hyperactivation	Tolomeo et al. (2022); Bösmüller et al. (2021); Zhang et al. (2022); Zhou et al. (2021)
p38	AVG(T1R, TLR, ROS)	TLR, angiotensin-II, and ROS leads to MAPK-p38 activation.	Grimes and Grimes (2020); Wang et al. (2018); Kaminskyy and Zhivotovsky (2014); Vollmer et al. (2017); Li et al. (2016)

mTORC1	AVG(NOT(TSC2), IKK)	TSC2 inhibits Rheb which promotes mTORC1 activation. IKKB activates mTORC1/2.	Feng and Levine (2010); Dan et al. (2016); Basile et al. (2022)
mTORC2	AVG(PI3K, AMPK, IKK)	PI3K promotes activation of mTORC2, AMPK promotes mTORC2 signaling. IKKB activates mTORC1/2.	Basile et al. (2022); Kazyken et al. (2019); Dan et al. (2016)
NF-κB	AVG(IKK, ROS) - FOXO3A	Phosphorylation of IκBs by IKK causes nuclear translocation of NF-κB, ROS activates NF-κB, absence of FOXO3A leads to activation of NF-κB	Hariharan et al. (2021); Kaminskyy and Zhivotovsky (2014); Guo et al. (2024); Cheema et al. (2021); Lee et al. (2008)
Nutr_Depr	Nutr_Depr	Nutrient Deprivation is an external factor	

p53	AVG(Hypoxia, AMPK, ROS) - Viral_Repl	Hypoxia induces p53, Metabolic stress via AMPK induces p53, SARS-CoV promotes RCHY1-mediated degradation of p53	Zhang et al. (2021); Labuschagne et al. (2018); Ma-Lauer et al. (2016); He et al. (2014); Koumenis et al. (2001); Budanov and Karin (2008)
PI3K	AVG(TLR, ROS, IL6R, IFNR)	TLR4 causes activation of PI3K, ROS promotes hyperphosphorylation of PI3K, IL6 trans-signalling leads to PI3K expression. Several type I IFNs (IFN-a, IFN-,b and IFN-w) induce activation of PI3K.	Laird et al. (2009); Koundouros and Poulogiannis (2018); Zegeye et al. (2018); Park et al. (2015); Qiu et al. (2020); Okoh et al. (2013); Zhu et al. (2022); Platanias (2005)
PTEN	p53	p53 enhances PTEN transcription	Jung et al. (2010)
RIG1	Viral_Repl	RIG-1 recognizes viral RNA	Yamada et al. (2021)

ROS	AVG(T1R, TLR) – AVG(FOXO3A, p53)	Binding of ANG II to AT1R causes ROS production, FOXO3A and p53 detoxifies ROS. TLR signaling promotes ROS generation.	Suhail et al. (2020); Covarrubias et al. (2008); Ferber et al. (2012); Ramasamy and Subbian (2021); Hwang et al. (2011); Hu et al. (2010); Suzuki et al. (2010); Zhang et al. (2011); Chen et al. (2009)
sIL6R	ADAM17	IL6R cleaved by ADAM17 to promote SIL6R	Magro (2020); Riethmueller et al. (2017); Stolarczyk et al. (2016)
STAT1	IFNR	IFNR activates STAT1	Matsuyama et al. (2020)
TLR	Virus	Viral proteins and RNA bind to TLR-2/3/4 among others	Mukherjee (2022)

TNF	AVG(ADAM17, NFKB, p38)	ADAM17, p38, and NFKB promote TNF-a	Hariharan et al. (2021); Campbell et al. (2004); Grimes and Grimes (2020); Mattos Pereira et al. (2024); Olímpio et al. (2023); Wong et al. (2005)
TNFR	TNF	TNF binds to its receptor	Mohd Zawawi et al. (2023)
TSC2	AVG(AMPK, HIF1a) - AKT	TSC2 is inhibited by Akt by phosphorylation, TSC2 is promoted by AMPK, HIF-1 induces REDD1 which releases TSC2 from inhibitory proteins, therefore promoting it.	Feng and Levine (2010); Dan et al. (2016); Budanov and Karin (2008); Brugarolas et al. (2004)
Viral_Repl	AVG(Virus, $(Virus\ \&\ mTORC1)$) - ISG	Viral Replication activated by virion, mTORC1 leads to Viral Proteins that promote Viral Replication, Viral Replication is inhibited by ISGs	Fattahi et al. (2022); Martin-Sancho et al. (2021)
Virus	Virus	Virus is an external factor	

This table does not show some of the inputs necessary to simulate some of the virtual experiments in section ??. These include TNF_e (external TNF), ANG2_e (external ANG2). These external inputs are incorporated in the model by an OR between them and the $f(target_i(s))$ (Eq. 1) function. I.e., TNFR = $OR(TNF\_e, f(AVG(ADAM17, NF\kappa B, p38))$ and $TR1 = OR(ANG2\_e, f(ANG2))$. This OR guarantees that adding the external TNF or ANG2 will activate the receptor.

Table S3: Changes made in the original model to fit it to the evaluation data. Column 1 output node. Column 2 target function. Column 3 description. Column 4 literature support. I bold we highlight the difference between the new target function and the original.

Node	target function	Change	Reference
ADAM17	AVG(T1R, **Virus**)	Virus activates ADAM17	Jiang et al. (2023)
AMPK	AVG(Nutr_Depr, p53, **!mTORC1**) - AKT	mTORC1 inhibits AMPK	Ling et al. (2020)
Apoptosis	AVG(CASP8, CASP9, **p53**)	TNF-a leads to apoptosis via p53; p53 is downstream of CASP8 and probably independent of CASP9	Sawada et al. (2004)
CASP9	AVG(FOXO3A, ROS) - BCL2	TNF-a leads to apoptosis via p53; p53 is downstream of CASP8 and probably independent of CASP9. (**p53 removed from the equation**).	Sawada et al. (2004)
p53	AVG(AMPK, **TNFR**) - Viral_Repl	TNF-a leads to apoptosis via p53; p53 is downstream of CASP8 and probably independent of CASP9 (**AMPK and ROS removed**)	Sawada et al. (2004)
ROS	AVG(T1R, TLR4, **Hypoxia**) – AVG(FOXO3A, p53)	Hypoxia creates ROS	Kanehisa Laboratories

Table S4 Target functions in the reduced model. Column 1 scenario (Scenarios 1-4). The labels in this column means: all: equations that are the same, independent of scenario. 2: Scenario 2. In this table, we choose to show the Scenario 2 equations for the Viral_repl and IFN, because they are the more "complete." Meaning that the Equations for the other scenarios are subsets of these equations. Scenarios A and B are artifacts of the reduced model. Because in the full model, different stress-related factors affect the mTOR pathway, both inhibitory and activating, in the reduced model, we end up with two alternating scenarios. Column 2 target node. Column 3 target function.

Scenario	Target	Equation
all	Virus	Virus
all	Inflammation	AVG(Virus, PI3K_AKT) - Stress
all	PI3K_AKT	AVG(Inflammation, Virus, IFNR) - Stress
all	Stress	Inflammation - AVG(Viral_repl, PI3K_AKT)
A	mTOR	AVG(PI3K_AKT, Inflammation) - Stress
B	mTOR	AVG(PI3K_AKT, Inflammation, Stress)
2	Viral_repl	AVG(Virus, (Virus & mTOR) - ISG)
all	IFNR	IFN
all	ISG	IFNR - Viral_repl
all	RIG1	Viral_repl
2	IFN	AVG(RIG1, Inflammation) - AVG(Viral_repl, mTOR)

Table S5: Table with the model independent evaluation data. Column 1, cell type. Column 2, experimental condition (e.g., infection with live virion, incubation with cytokine). Column 3, Time of the measurement in hours. Column 4, Node queried. Protein/phosphoprotein or other quantity measured experimentally, which is also present in the Network. Column 5, experimental measurement. Possibilities are up or down in relation to control (i.e., without stimuli). Columns 6-9, predicted value by scenarios 1-4. Column 10, reference for data in columns 2-5. Blue: up. Red: down. * transgenic A549 cells expressing ACE2 receptor.

cell	Condition	time	measured quantity	obs	1	2	3	4	Ref
Calu-3	SARS-CoV-2	48	CASP-8 (cleaved-Casp-8)	up	up	up	up	up	Li et al. (2020)
Calu-3	SARS-CoV-2	48	CASP-9 (cleaved-casp-9)	up	up	up	up	up	Li et al. (2020)
Calu-3	SARS-CoV-2	24	IFN-I	up	up	up	up	no	Rebendenne et al. (2021)
Calu-3	SARS-CoV-2	48	IL6	no	up	up	up	up	Rebendenne et al. (2021)
Calu-3	SARS-CoV-2	24	ISG (ISG15)	up	up	up	no	no	Saccon et al. (2021)
Calu-3	SARS-CoV-2	48	p-IRF3	up	up	up	up	up	Jiang et al. (2021)
Calu-3	SARS-CoV-2	48	p-p38	up	up	up	up	up	Jiang et al. (2021)

Calu-3	SARS-CoV-2	24	p-STAT1	up	up	up	up	no	Saccon et al. (2021)
Calu-3	SARS-CoV-2	24	RIG-I	up	up	up	up	up	Saccon et al. (2021)
Calu-3	SARS-CoV-2	48	TNF	up	up	up	up	up	Rebendenne et al. (2021)
A549*	SARS-CoV-2	24	HIF1a	up	up	up	up	up	Eltzschig et al. (2023)
A549*	SARS-CoV-2	48	p-NFkB	up	up	up	up	up	Patra et al. (2020)
A549*	SARS-CoV-2	48	ADAM-17	up	no	no	no	no	Patra et al. (2020)
Calu-3	viral protein	24	IL6	up	up	up	up	up	Khan et al. (2021)
Calu-3	viral protein	24	TNF	up	up	up	up	up	Khan et al. (2021)
A549	viral protein	24	IL6	up	up	up	up	up	Zhang et al. (2007)
A549	viral protein	24	p-NFkb	up	up	up	up	up	Zhang et al. (2007)
A549*	viral protein	24	p-STAT1	no	up	up	up	up	Zhang et al. (2007)
A549*	viral protein	24	TNF	no	up	up	up	up	Forsyth et al. (2022)
A549*	viral protein	48	ADAM-17	up	no	no	no	no	Patra et al. (2020)
A549*	viral protein	48	ACE2	no	no	no	no	no	Patra et al. (2020)
A549*	viral protein	24	p-AKT	up	up	up	up	up	Arjsri et al. (2022)
A549	hypoxia	0.5-1	HIF1a	up	up	up	up	up	Ushijima et al. (2015)
A549	hypoxia	0.5-1	mTORC1 (p-p70S6K)	no	no	no	no	no	Ushijima et al. (2015)
A549	hypoxia	1-16h	p-p53	no	up	up	up	up	Sermeus et al. (2013)
A549	hypoxia	48	p-p38	up	no	no	no	no	Yadav et al. (2017)
A549	hypoxia	48	BCL-2	no	up	up	up	up	Yadav et al. (2017)
A549	gluc starvation	24	p-AMPK	up	up	up	up	up	Li et al. (2020)
A549	gluc starvation	24	p-AKT	no	up	up	up	up	Li et al. (2020)
A549	gluc starvation	24	p-AMPK	no	up	up	up	up	Püschel et al. (2020)
A549	gluc starvation	24	IL6	no	up	up	up	up	Püschel et al. (2020)
A549	gluc starvation	24	mTORC1 (p-S6)	no	no	no	no	no	Püschel et al. (2020)
A549	starvation	1	FOXO3A (nucl transloc)	up	up	up	up	up	Uliczka et al. (2024)
Calu-3	Poli I:C/ HIF1a RNAi	24	IL6	up	up	up	up	up	Polke et al. (2017)
A549	TNF	24	*cFLIP*	up	up	up	up	up	Ooppachai et al. (2019)
A549	TNF	24	CASP-9 (cleaved Casp-9)	no	no	no	no	no	Ooppachai et al. (2019)
A549	TNF	24	CASP-8 (cleaved Casp-8)	no	up	up	up	up	Ooppachai et al. (2019)
A549	TNF	24	Apoptosis	up	up	up	up	up	Ooppachai et al. (2019)
A549	TNF	24	*IL6*	up	up	up	up	up	Chen et al. (2020)
A549	TNF	24	*NFKB (p-p65)*	up	up	up	up	up	Chen et al. (2020)

A549	TNT	24	*acetyl-p53*	up	no	no	no	no	Chen et al. (2020)
A549	TNF	24-48	*BCL-2*	no	up	up	up	up	Li et al. (2013)
A549	ANG-II	20	Apoptosis	up	up	up	up	up	Papp et al. (2002)
A549	ANG-II	24	*ACE2*	no	no	no	no	no	Wang et al. (2024)
A549	ANG-II	24	*ADAM-17*	up	up	up	up	up	Wang et al. (2024)
A549	ANG-II	24	*p-NFKB*	up	up	up	up	up	Wang et al. (2024)
A549	ANG-II	24	*TNF*	up	up	up	up	up	Wang et al. (2024)
A549	ANG-II	24	*IL6*	up	up	up	up	up	Wang et al. (2024)
A549	Sirolimus	48	p-p53	no	no	no	no	no	Krześniak et al. (2014)
A549	Sirolimus	48	p-AKT	up	no	no	no	no	Krześniak et al. (2014)
A549	Sirolimus	48	Apoptosis	up	no	no	no	no	Shin et al. (2021)
A549	Sirolimus	24	p-AKT	no	no	no	no	no	Chen et al. (2015)
A549	Sirolimus	24	mTORC1 (p-4E-BP1)	no	no	no	no	no	Chen et al. (2015)
A549	Sirolimus	24	p-FOXO3A	up	no	no	no	no	Chen et al. (2015)
A549	Sirolimus	21	p-AMPK	up	no	no	no	no	Alhasan et al. (2024)
A549	Sirolimus	24	mTORC1 (p-p70S6K)	no	no	no	no	no	Shin et al. (2021)

Table S6: Table showing the interactions that existed in the original model and the expansion with Wikipathways present in the COVID-19 Disease Map (University of Luxembourg (2026); the COVID-19 Disease Map Community et al. (2021)). Column 1 shows the target node, Column 2 the source node, Column three weather that interaction was positive (activatory) or negative (inhibitory), Column 4 whether that interaction was already present in the original model (yes) or was present only in the COVID-19 Disease Map (no), Column 4 shows the Wikipathways that suport that interaction (if any), and Column 5 tells if that interaction was added to the model or not. Possible values are NA (Not apply), Yes, and No. NA means that the interaction was present in both the Wikipathway and the original model; therefore, there was no need to add, or that it was an interaction related to Apoptosis. Because the reduced model was carved only to reproduce the experiment from Figure **??**, and Apoptosis does not influence those outcomes, we did not implement that pathway. Wikipathways interactions were refused for three reasons: 1. TLR→NFκB was refused because it was redundant with TLR→IKK→NFκB. Besides, we already tested that in Figure S4. 2: Viral_repl ⊣ RIG1 was refused because we can not, with a Boolean network, both inhibit and activate the same target, as the pathways WP4880 and WP4868 show. 3: Viral_repl inhibition of STAT1 and IRF3 were refused. We were aware of those inhibitions when we built the model. We chose not to add them. Instead, we chose to represent only Viral_repl ⊣ ISG and Viral_repl ⊣ IFN; the others are redundant.

**Target**	**Source**	**Interaction Type**	**In The Original Model**	**Wikipathways**	**Added To The Model**
ACE	ACE	positive	yes		
ACE	Virus	negative	yes	WP4912, WP4799, WP4883	NA
ACE	ADAM	negative	yes		
ADAM	T1R	positive	yes		
AKT	mTORC2	positive	yes		
AKT	IKK	positive	yes		
AKT	PI3K	positive	yes		
AKT	PTEN	negative	yes		
AKT	Viral_repl	negative	no	WP4864	Yes
AMPK	Nutr_depr	positive	yes		
AMPK	p53	positive	yes		
AMPK	AKT	negative	yes		
ANG2	ACE2	negative	yes	WP4799, WP4883	NA
T1R	ANG2	positive	yes	WP4799, WP4883	NA
apoptosis	CASP8	positive	yes	WP4864	NA

apoptosis	CASP9	positive	yes	WP4864	NA
apoptosis	AKT	negative	no	WP4864	NA
apoptosis	p38	positive	no	WP4864	NA
apoptosis	Viral_repl	positive	no	WP4864	NA
BCL2	NFKB	positive	yes		
BCL2	CREB1	positive	yes		
BCL2	p53	negative	yes		
BLC2	Viral_repl	negative	no	WP4864	NA
CASP8	FADD	positive	yes	WP4864	
CASP8	p53	positive	yes		
CASP8	c-FLIP	negative	yes		
CASP9	FOXO3A	positive	yes		
CASP9	p53	positive	yes		
CASP9	ROS	positive	yes		
CASP9	BCL2	negative	yes		
c-FLIP	NFKB	positive	yes		
c-FLIP	p53	positive	yes		
c-FLIP	AKT	negative	yes		
CREB1	AKT	positive	yes		
CREB1	Viral_repl	positive	yes		
FADD	TNF	positive	yes		
FOXO3A	p38	positive	yes		
FOXO3A	AMPK	positive	yes		
FOXO3A	IKK	negative	yes		
FOXO3A	AKT	negative	yes		
HIF1A	ROS	positive	yes		
HIF1A	Hypoxia	positive	yes		
HIF1A	mTORC1	positive	yes		
IFN	IRF3	positive	yes	WP4880, WP4868	NA
IFN	NFKB	positive	no	WP4880	Yes
IFN	p38	positive	no	WP4880	Yes
IFN	FOXO3A	positive	yes		
IFN	Viral_repl	negative	yes	WP4868	NA
IFNR	IFN	positive	yes	WP4880, WP4868, WP4912	NA
IL6	NFKB	positive	yes		
IL6	p38	positive	yes		
IL6R	IL6	positive	yes		
IRF3	RIG1	positive	yes	WP4880, WP4868, WP4912	NA
IRF3	TLR	positive	no	WP4880, WP4868, WP4912	Yes
IRF3	Viral_repl	negative	no	WP4880, WP4868	No
IKK	TLR	positive	yes		
IKK	TNFR	positive	yes		
IKK	AKT	positive	yes		

ISG	STAT1	positive	yes	WP4880, WP4868	NA
ISG	Viral_repl	negative	yes	WP4880	NA
p38	T1R	positive	yes		
p38	TLR	positive	yes		
p38	ROS	positive	yes		
p38	Viral_repl	negative	yes	WP4880	NA
mTORC1	TSC2	negative	yes		
mTORC1	IKK	positive	yes		
mTORC2	PI3K	positive	yes		
mTORC2	AMPK	positive	yes		
mTORC2	IKK	positive	yes		
NFKB	IKK	positive	yes		
NFKB	ROS	positive	yes		
NFKB	FOXO3A	negative	yes		
NFKB	Viral_repl	negative	no	WP4880	Yes
NFKB	TLR	positive	no	WP4880, WP4868	No
p53	Hypoxia	positive	yes		
p53	AMPK	positive	yes		
p53	ROS	positive	yes		
p53	Viral_repl	negative	yes		
PI3K	TLR	positive	yes		
PI3K	ROS	positive	yes		
PI3K	IL6R	positive	yes		
PI3K	IFNR	positive	yes		
PTEN	p53	positive	yes		
RIG1	Viral_repl	positive	yes	WP4880, WP4868, WP4912	NA
RIG1	Viral_repl	negative	no	WP4880, WP4868	No
ROS	T1R	positive	yes		
ROS	TLR	positive	yes		
ROS	FOXO3A	negative	yes		
ROS	p53	negative	yes		
sIL6R	ADAM	positive	yes		
STAT1	IFNR	positive	yes	WP4880, WP4868, WP4912	NA
STAT1	Viral_repl	negative	no	WP4880, WP4868	No
TLR	Virus	positive	yes		
TNF	ADAM	positive	yes		
TNF	NFKB	positive	yes		
TNF	p38	positive	yes		
TNFR	TNF	positive	yes		
TSC2	AMPK	positive	yes		
TSC2	HIF1A	positive	yes		
TSC2	AKT	negative	yes		
Viral_repl	Virus	positive	yes		

Viral_repl	mTORC1	positive	yes
Viral_repl	ISG	negative	yes

2 Appendix B: Supplementary Methods

2.1 Model

In this work, we use the framework of **Schaub** (**Schaub et al.** (**2007**)). Let c_i be a Boolean network component belonging to the set C of network components (Where $|C| = k$). Each component can have one of two states: 1: active; 0: inactive. The state of the whole network "s" corresponds to the vector $(c_0, ..., c_k) \in \{0,1\}^k$ of states of all components. For each c_i, there exists a target function $target_i \in T$ that specifies the value to which it should move ($target_i : \{0,1\}^n \rightarrow \{0,1\}$). These target functions are the actual Boolean or Qualitative Equations. In our model they can be seen on Tables S2 and S3. The Equation below shows how to compute the value for each component in the next time step:

$$c_i = f_i(target_i(s)) \tag{1}$$

and

$$f_i(target_i(s)) = \begin{cases} 1 \text{ if } target_i(s) > c_i \\ 0 \text{ if } target_i(s) < c_i \\ c_i \text{ if } target_i(s) = c_i \end{cases}$$

Where the target functions are given in tables S2 and S3, but are usually of the form:

$$target_i(s) = AVG_i(c_{act}) - AVG_i(c_{inh}) = \sum_{a_{ji=1}} \frac{a_{ji} \times c_j}{a_{ji}} - \sum_{a_{ji=-1}} \frac{a_{ji} \times c_j}{a_{ji}}$$

Where a_{ji} is the weight of the arc between i and j. In our case we assume it to be always one for simplicity and due to the lack of data to fit those weights. The model above is agnostic to the update order, and in our work, we use an asynchronous stochastic update order.

The rationale for using averaged activation functions is that traditional Boolean operators fail to capture important biological nuance. Consider a node $z = f(x, y)$, where z is regulated by two upstream nodes x and y. If $f(...)$ is an OR operator, activation of either x or y is sufficient to fully activate z. However, in many biological systems, when a gene or protein has multiple activators, the effect of a single activator is typically weaker than the combined effect of both acting together (see, for example some of the cases shown in **Schaub** (**Schaub et al.** (**2007**))). Conversely, if $f(...)$ is an AND operator, both x and y must be active for z to activate, whereas in practice either activator alone may be sufficient to induce partial activation, with full activation emerging only when both are present.

Averaging-based update rules provide a natural resolution to this limitation. Taking the average of x and y yields full activation if and only if both inputs are active, while producing intermediate activation levels when only one input is active. In this way, averaging captures graded responses without imposing rigid logical thresholds. Such partial activations can be instantiated in two complementary ways. In the classical multivalued Boolean formalism proposed by **Schaub (Schaub et al. (2007))**, nodes take values in the discrete range $\{0, \ldots, N\}$ with $N > 1$; for example, 0 denotes inactivity, 1 partial activation, and 2 full activation. In contrast, the approach adopted here represents the same logic using an ensemble of binary $0, 1$ networks with stochastic, asynchronous updates. While each individual network exhibits only binary states, the ensemble average yields continuous activation levels between 0 and 1. In this representation, z=1 corresponds to full activation, z=0 to inactivity, and $0 < z < 1$ to partial activation.

This approach is able to reproduce key behaviors addressed by the multivalued formalism shown in **Schaub (Schaub et al. (2007))** (not shown), while naturally introducing variability across realizations. This increased variance is biologically realistic, reflecting cell-to-cell heterogeneity, albeit at the cost of some of the sharp, black-and-white predictions characteristic of traditional Boolean models.

2.2 When Did We Used Traditional Boolean Functions?

The explanation that follows applies only to Tables S2. Table S3 is considered fitting (adjustment); therefore, we are free to choose the function that best fits the data. The AND was used only once, in the viral-replication equation: "Virus & mTORC1" (the full equation is Viral_repl = AVG(Virus, (Virus & mTORC1)) - ISG). We have to put an AND here because otherwise, uninfected cells would produce viral particles just because mTORC1 is active.

NOT is used two times. "ANG2 = !ACE2." Here, the lack of ACE2 leads to the "production" (or accumulation) of ANG2. However, if the equation were "ANG2 = -ACE2", then "ANG2 = 1" would hold only if ANG2 were already equal to 1. That is, ANG2 accumulation would not be possible. The second is in the mTORC1 equation "!TSC2" (the full equation is mTORC1 = AVG(!TSC2, IKK)). This one is a matter of nuance, and it also has to be this way since the literature considers that the lack of TSC2 is sufficient to activate mTORC1. If we wrote the equation "IKK - TSC2", TSC2=0 would lead to mTORC1=1 if and only if mTORC1 were already equal to one. But that is not what the literature says. The literature says that once you remove TSC2, mTORC1 becomes active.

We only use OR to deal with external inputs. For example, external TNF and external ANG2. Those are external sources of these molecules, different from the ones produced by the network. The external ones are used to simulate incubation with TNF or angiotensin-II. The OR here is a simplified way to guarantee that these external sources will activate their receptors. **It operate outside Equation 1** and are not shown in Tables S2 and S3. Except when we introduce an external input, which was done only when we compared the model with an independent dataset (section **??**), these OR operators can be ignored.

2.3 Determining Convergency

We compared a sliding window of six iterations with the last two iterations of the simulations across all nodes that had variance bigger than zero (I.e., those whose values changed during the simulation) and across 400 random networks shuffled with the methods previously described. We took care not to slide the sliding window so that the last two iterations would be inside it. We compared this sliding window with respect to variance and average. Figure S9 shows that for the initial iterations, the percentage of p-values below 0.05 was substantially higher than 5%, while this value quickly converged to close to 5%, with the last three bars in Figure S9A ranging from 4.8-5.3% and the last three bars in Figure S9B ranging between 5.4-5.5%. Under an alpha of 0.05, it is expected that 5% of p-values will be below 0.05 just by chance. We also tested a window size of two and obtained similar results (not shown). This shows that the average and variance of the last six to eighteen iterations of the Network seems to be indistinguishable from those of the last two iterations.

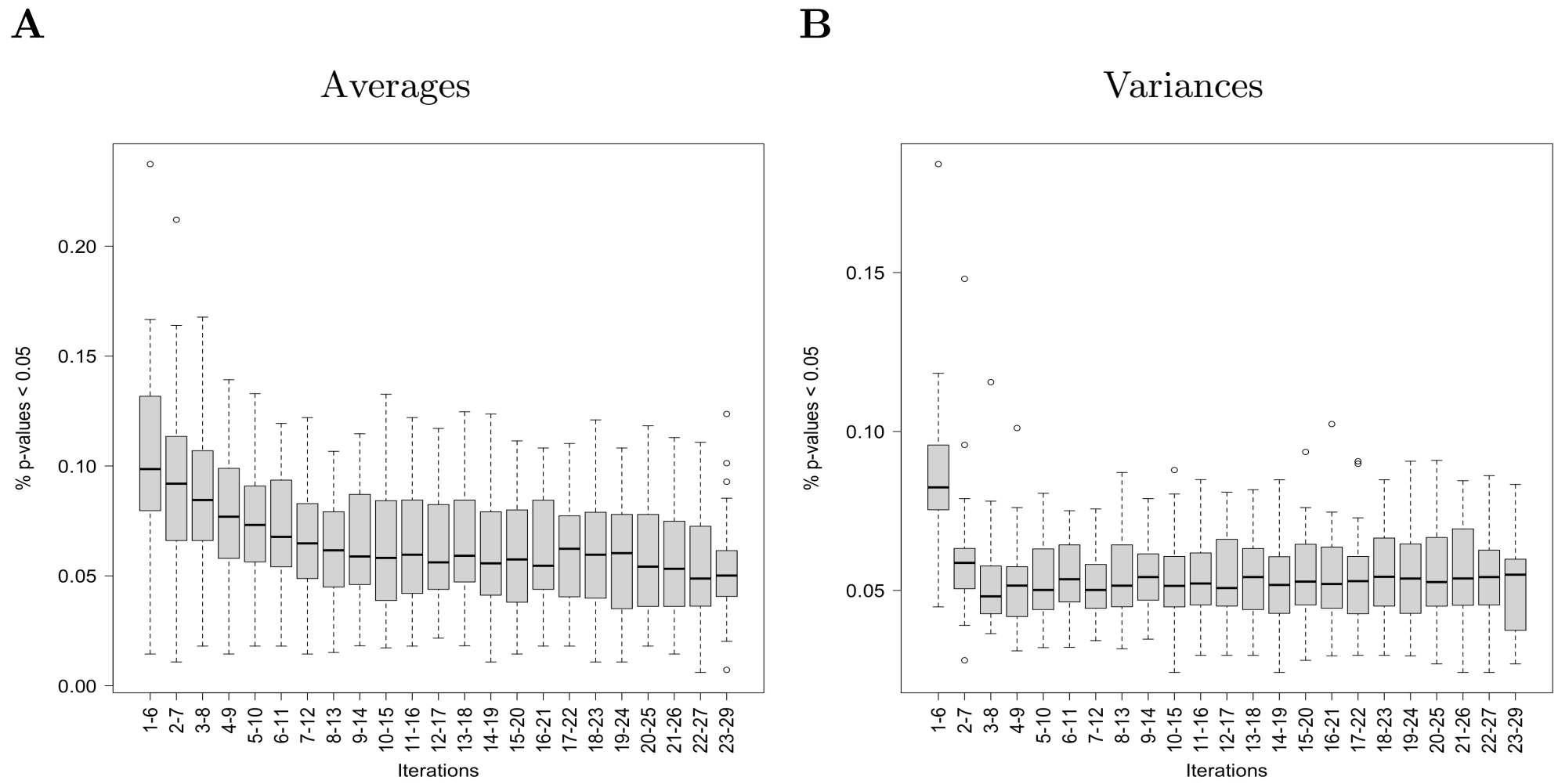

Fig. S9 Graphics showing the convergence of the networks. We generated 400 random network realizations initialized from scenario 2, the most complete model configuration, and compared a sliding window of size six iterations with the last two iterations of each node. We compared the mean of the sliding window with the mean of the last two iterations using Student's t-test and the variance using Fisher's test. Each bar is the aggregate of 400 simulations and 23 nodes. While the Network has more than 23 nodes, only 23 exhibited changes during the simulations, with the others remaining with a fixed value (usually zero) across the simulation. After the test (t-test: A or F-test: B), we counted the number of times the test had fallen below 0.05. The expected by chance is 5%. A: Comparison of the averages. B: Comparison of the variances.

2.4 Simulations

We performed all model simulations using random asynchronous update orders. To simulate cells without any stimuli, we initialized the networks with ACE2=1, TSC2=1,

and all other variables set to 0 (Algorithm 2). To simulate a cell infected with SARS-CoV-2, we first ran 30 iterations of resting cells and then initialized the appropriate input with one (*e.g.*, Virus=1 to simulate infected cells – See algorithms 1 and 2). By an extensive check of many different simulations, we found that 15 iterations were enough for them to reach a steady state, and for safety, we ran 30.

Algorithm 1 Procedure to run the Boolean network asynchronously and return the last two iterations' aggregate.

function RunBN($\mathbf{s}, n$)
    for $i \leftarrow 0$ **to** 30 **do**
        $\mathbf{r} \leftarrow \mathbf{s}$
        $\mathbf{L} \leftarrow$ RandomPermutation($1..n$)
        for all $j \in L$ **do**
            $c_j \leftarrow f_j(\mathbf{s})$
        end for
    end for
    $\mathbf{s} = \mathbf{s} \vee \mathbf{r}$
    return s
end function

Algorithm 2 Basic procedure used to run the network. All experiments were variations of this algorithm. Here, n is the network size, K is the ensemble size, and E is the ensemble. We opted to use a specific example here for stimulation, *i.e.*, viral infection.

$n \leftarrow 52$
$K \leftarrow 500$
$\mathbf{E_{rest}} \in \mathbb{R}^n \leftarrow \mathbf{0}$
$\mathbf{E_{stimul}} \in \mathbb{R}^n \leftarrow \mathbf{0}$
for $i \leftarrow 0$ **to** K **do**
    $\mathbf{s} \in \mathbb{N}^n \leftarrow \mathbf{0}$
    $\mathbf{s_{TSC2}} \leftarrow 1$
    $\mathbf{s_{ACE2}} \leftarrow 1$
    $\mathbf{s} \leftarrow$ RunBN($\mathbf{s}, n$)
    $\mathbf{E_{rest}} \leftarrow \mathbf{E_{rest}} + \mathbf{s}$
    $\mathbf{s_{virus}} \leftarrow 1$
    $\mathbf{s} \leftarrow$ RunBN($\mathbf{s}, n$)
    $\mathbf{E_{stimul}} \leftarrow \mathbf{E_{stimul}} + \mathbf{s}$
end for
$\mathbf{E_{rest}} \leftarrow \mathbf{E_{rest}}/K$
$\mathbf{E_{stimul}} \leftarrow \mathbf{E_{stimul}}/K$
$\mathbf{E_{stimul}} \leftarrow \mathbf{E_{stimul}} - \mathbf{E_{rest}}$

Due to the asynchronous update, the behavior of the model changes from simulation to simulation. Therefore, we ran ensembles of 500 different update orders and computed the average. In Figure S2, we see the result of ensembles of models.

Algorithm 2 shows the basic process of model execution. We run 30 iterations of resting cells, add the disjunction of its last two state vectors to an ensemble, update that last state vector with a signal (in the algorithm virus), run another 30 iterations, and add the last two state vectors to another ensemble. Finally, update the stimulated ensemble by subtracting the resting ensemble. We worked with the last two iterations as an imperfect but simple method to avoid the effect of oscillations.

The number of simulations in the ensemble was a trade-off between computational cost and statistical power. In general, the number of simulations in the ensemble does not change the averages, but the standard deviation decreases asymptotically to zero as the ensemble size grows to infinity.

2.5 Determining if a Node is Active

The final ensemble of models has values in the real domain $[-1, 1]$, which we interpret as upregulated/active if positive and downregulated/inactive/unchanged otherwise. We considered a node positive if its average value minus 1.96 times its standard error was greater than zero. We ran multiple ensembles and computed the average of averages and the standard deviation of averages. The average of averages is just a more accurate estimate of it, while the standard deviation of the averages is the standard error. Each ensemble consisted of 500 models, as already mentioned, while for the evaluation, we ran thirty batches of ensembles to compute statistics.

2.6 Perturbation Analysis

There are two types of related perturbations that we considered in this analysis: the knock-out of a connection and the knock-in of a connection. The first is the removal, while the second is the introduction of an edge that did not exist before. For the knock-out, all 96 edges in the model (see Figure 2) were eligible for deletion. However, the knock-in is more complicated because, in addition to the two nodes being connected, we also need to choose the direction and type of connection (*i.e.*, activation, inhibition). Therefore, we resorted to KEGG pathways and selected interactions that were present in their maps but not in our model, which yielded a list of 16 edges with directionality and their type (inhibition or activation).

It would be convenient to treat the knock-outs and knock-ins as subclasses of perturbations. Conversely, we will define a perturbation here as a knock-out or a knock-in. We have a list of 112 possible perturbations (*i.e.*, 96 knock-outs + 16 knock-ins). We performed these perturbations one at a time and two at a time. This gave us a list of 6,328 variant models (see the equation below) for each scenario. Therefore, we have a total of 25,312 variants.

$$112 + \binom{112}{2} = 112 + \frac{112 \times 112}{2} = 6328$$

2.6.1 Robustness of the four scenarios

The perturbation analysis described before was used to assess the robustness of the four scenarios. In this study, we repeated the type of analysis used to generate Figure 5. Briefly, we generated 100 replicates of IFN and viral-replication levels/expression for models initialized with virus and with virus and Sirolimus, for each of the 25,312 model variants (*i.e.*, 6,328 in each scenario). We then counted the number of times Sirolimus-treated simulations had higher IFN and lower viral replication than non-treated simulations, with $p < 10^{-5}$. A caveat of this analysis was that it was computationally intensive, and to allow for suitable execution time, we decreased the ensembles from 500 to 100.

2.6.2 Vertex Knock-out

The perturbation analysis described previously was also used to assess how a list of twelve central nodes affects viral replication. We selected the twelve nodes based on their centrality and pathway representativeness (more on this below). No apoptosis node was selected because this pathway does not affect viral replication in this model. In this analysis, for each of the 25,312 model variants, we knocked out the twelve critical nodes one at a time. We then measured the number of times it led to an increase or decrease in the four outputs of more than 5% compared to the same model variant without the vertex knock-out. We also used ensembles of 100 for consistency with the previous analysis.

The twelve nodes were chosen first based on their degree centrality (in-degree + out-degree). We collected all nodes with a degree (in+out) of seven or greater (AKT, FOXO3A, IKK, NfκB, p53, ROS). Then we added T1R and ADAM-17 as representatives of the renin-angiotensin pathway. Which in the model is collapsed with the inflammation pathway. We added AMPK for its known relevance as an upstream factor of mTORC1. We added IFNR as a representative of IFN/STAT1, without which no IFN signaling happens. We added p38 due to its known relevance to inflammation, and finally, TORC1, which is the main factor being studied in this paper.

References

Arora, S., Ahmad, S., Irshad, R., Goyal, Y., Rafat, S., Siddiqui, N., Dev, K., Husain, M., Ali, S., Mohan, A., Syed, M.A.: Tlrs in pulmonary diseases. Life sciences **233**, 116671 (2019)

Alhasan, B., Gladova, Y.A., Sverchinsky, D.V., Aksenov, N.D., Margulis, B.A., Guzhova, I.V.: Hsp70 negatively regulates autophagy via governing ampk activation, and dual hsp70-autophagy inhibition induces synergetic cell death in nsclc cells. International journal of molecular sciences **25**, 9090 (2024)

Arjsri, P., Srisawad, K., Mapoung, S., Semmarath, W., Thippraphan, P., Umsumarng, S., Yodkeeree, S., Dejkriengkraikul, P.: Hesperetin from root extract of

¡i¿clerodendrum petasites¡/i¿ s. moore inhibits sars-cov-2 spike protein s1 subunit-induced nlrp3 inflammasome in a549 lung cells via modulation of the akt/mapk/ap-1 pathway. International journal of molecular sciences **23**, 10346 (2022)

Basile, M.S., Cavalli, E., McCubrey, J., Hernández-Bello, J., Muñoz-Valle, J.F., Fagone, P., Nicoletti, F.: The pi3k/akt/mtor pathway: A potential pharmacological target in covid-19. Drug discovery today **27**, 848–856 (2022)

Babaev, V.R., Ding, L., Zhang, Y., May, J.M., Lin, P.C., Fazio, S., Linton, M.F.: Macrophage ikkα deficiency suppresses akt phosphorylation, reduces cell survival, and decreases early atherosclerosis. Arteriosclerosis, thrombosis, and vascular biology **36**, 598–607 (2016)

Budanov, A.V., Karin, M.: p53 target genes sestrin1 and sestrin2 connect genotoxic stress and mtor signaling. Cell **134**, 451–60 (2008)

Brugarolas, J., Lei, K., Hurley, R.L., Manning, B.D., Reiling, J.H., Hafen, E., Witters, L.A., Ellisen, L.W., Kaelin, W.G.: Regulation of mtor function in response to hypoxia by redd1 and the tsc1/tsc2 tumor suppressor complex. Genes & development **18**, 2893–904 (2004)

Bösmüller, H., Matter, M., Fend, F., Tzankov, A.: The pulmonary pathology of covid-19. Virchows Archiv : an international journal of pathology **478**, 137–150 (2021)

Brasier, A.R.: The nuclear factor-kappab-interleukin-6 signalling pathway mediating vascular inflammation. Cardiovascular research **86**, 211–8 (2010)

Bartke, T., Siegmund, D., Peters, N., Reichwein, M., Henkler, F., Scheurich, P., Wajant, H.: p53 upregulates cflip, inhibits transcription of nf-kappab-regulated genes and induces caspase-8-independent cell death in dld-1 cells. Oncogene **20**, 571–80 (2001)

Campbell, J., Ciesielski, C.J., Hunt, A.E., Horwood, N.J., Beech, J.T., Hayes, L.A., Denys, A., Feldmann, M., Brennan, F.M., Foxwell, B.M.J.: A novel mechanism for tnf-alpha regulation by p38 mapk: involvement of nf-kappa b with implications for therapy in rheumatoid arthritis. Journal of immunology (Baltimore, Md. : 1950) **173**, 6928–37 (2004)

Chen, M., Chen, Z., Huang, D., Sun, C., Xie, J., Chen, T., Zhao, X., Huang, Y., Li, D., Wu, B., Wu, D.: Myricetin inhibits tnf-α-induced inflammation in a549 cells via the sirt1/nf-κb pathway. Pulmonary pharmacology & therapeutics **65**, 102000 (2020)

Chaudhary, P.M., Eby, M., Jasmin, A., Bookwalter, A., Murray, J., Hood, L.: Death receptor 5, a new member of the tnfr family, and dr4 induce fadd-dependent apoptosis and activate the nf-kappab pathway. Immunity **7**, 821–30 (1997)

Cha, S.-I., Groshong, S.D., Frankel, S.K., Edelman, B.L., Cosgrove, G.P., Terry-Powers, J.L., Remigio, L.K., Curran-Everett, D., Brown, K.K., Cool, C.D., Riches, D.W.H.: Compartmentalized expression of c-flip in lung tissues of patients with idiopathic pulmonary fibrosis. American journal of respiratory cell and molecular biology **42**, 140–8 (2010)

Covarrubias, L., Hernández-García, D., Schnabel, D., Salas-Vidal, E., Castro-Obregón, S.: Function of reactive oxygen species during animal development: passive or active? Developmental biology **320**, 1–11 (2008)

Cheema, P.S., Nandi, D., Nag, A.: Exploring the therapeutic potential of forkhead box o for outfoxing covid-19. Open biology **11**, 210069 (2021)

Cohen, G.M.: Caspases: the executioners of apoptosis. The Biochemical journal **326 (Pt 1)**, 1–16 (1997)

COVID-19 Disease Map. https://covid.pages.uni.lu/. an online knowledge repository of molecular mechanisms of COVID-19, community-driven effort to assemble virus–host interaction diagrams based on literature evidence (2026)

Carracedo, A., Pandolfi, P.P.: The pten-pi3k pathway: of feedbacks and cross-talks. Oncogene **27**, 5527–41 (2008)

Chen, W., Sun, Z., Wang, X.-J., Jiang, T., Huang, Z., Fang, D., Zhang, D.D.: Direct interaction between nrf2 and p21(cip1/waf1) upregulates the nrf2-mediated antioxidant response. Molecular cell **34**, 663–73 (2009)

Chen, B., Xu, X., Luo, J., Wang, H., Zhou, S.: Rapamycin enhances the anti-cancer effect of dasatinib by suppressing src/pi3k/mtor pathway in nsclc cells. PloS one **10**, 0129663 (2015)

Dan, H.C., Antonia, R.J., Baldwin, A.S.: Pi3k/akt promotes feedforward mtorc2 activation through ikkα. Oncotarget **7**, 21064–75 (2016)

Du, K., Montminy, M.: Creb is a regulatory target for the protein kinase akt/pkb. The Journal of biological chemistry **273**, 32377–9 (1998)

Das, T.P., Suman, S., Alatassi, H., Ankem, M.K., Damodaran, C.: Inhibition of akt promotes foxo3a-dependent apoptosis in prostate cancer. Cell death & disease **7**, 2111 (2016)

Essafi, A., Mattos, S., Hassen, Y.A.M., Soeiro, I., Mufti, G.J., Thomas, N.S.B., Medema, R.H., Lam, E.W.-F.: Direct transcriptional regulation of bim by foxo3a mediates sti571-induced apoptosis in bcr-abl-expressing cells. Oncogene **24**, 2317–29 (2005)

Eltzschig, H., Yuan, X., Kim, J., Wang, Y., Mangala, L., Rodriguez-Aguayo, C.,

Lopez-Berestein, G., Sood, A., Ruan, W., Weng, T., et al.: Hif-dependent induction of alveolar mir-147 dampens sars-cov-2 immune evasion (2023) https://doi.org/10.21203/rs.3.rs-2319034/v1

Fattahi, S., Khalifehzadeh-Esfahani, Z., Mohammad-Rezaei, M., Mafi, S., Jafarinia, M.: Pi3k/akt/mtor pathway: a potential target for anti-sars-cov-2 therapy. Immunologic research **70**, 269–275 (2022)

Feng, Z., Levine, A.J.: The regulation of energy metabolism and the igf-1/mtor pathways by the p53 protein. Trends in cell biology **20**, 427–34 (2010)

Ferber, E.C., Peck, B., Delpuech, O., Bell, G.P., East, P., Schulze, A.: Foxo3a regulates reactive oxygen metabolism by inhibiting mitochondrial gene expression. Cell death and differentiation **19**, 968–79 (2012)

Forsyth, C.B., Zhang, L., Bhushan, A., Swanson, B., Zhang, L., Mamede, J.I., Voigt, R.M., Shaikh, M., Engen, P.A., Keshavarzian, A.: The sars-cov-2 s1 spike protein promotes mapk and nf-kb activation in human lung cells and inflammatory cytokine production in human lung and intestinal epithelial cells. Microorganisms **10**, 1996 (2022)

García-Cuellar, C.M., Chirino, Y.I., Morales-Bárcenas, R., Soto-Reyes, E., Quintana-Belmares, R., Santibáñez-Andrade, M., Sánchez-Pérez, Y.: Airborne particulate matter (pm_{10}) inhibits apoptosis through pi3k/akt/foxo3a pathway in lung epithelial cells: The role of a second oxidant stimulus. International journal of molecular sciences **21**, 473 (2020)

Grimes, J.M., Grimes, K.V.: p38 mapk inhibition: A promising therapeutic approach for covid-19. Journal of molecular and cellular cardiology **144**, 63–65 (2020)

Guo, Q., Jin, Y., Chen, X., Ye, X., Shen, X., Lin, M., Zeng, C., Zhou, T., Zhang, J.: Nf-κb in biology and targeted therapy: new insights and translational implications. Signal transduction and targeted therapy **9**, 53 (2024)

Gimenes-Junior, J., Owuar, N., Vari, H.R., Li, W., Xander, N., Kotnala, S., Sajjan, U.S.: Foxo3a regulates rhinovirus-induced innate immune responses in airway epithelial cells. Scientific reports **9**, 18180 (2019)

Huang, W.-C., Hung, M.-C.: Beyond nf-κb activation: nuclear functions of iκb kinase α. Journal of biomedical science **20**, 3 (2013)

Hariharan, A., Hakeem, A.R., Radhakrishnan, S., Reddy, M.S., Rela, M.: The role and therapeutic potential of nf-kappa-b pathway in severe covid-19 patients. Inflammopharmacology **29**, 91–100 (2021)

Hu, M.C.-T., Lee, D.-F., Xia, W., Golfman, L.S., Ou-Yang, F., Yang, J.-Y., Zou, Y., Bao, S., Hanada, N., Saso, H., Kobayashi, R., Hung, M.-C.: Ikappab kinase promotes

tumorigenesis through inhibition of forkhead foxo3a. Cell **117**, 225–37 (2004)

Hawley, S.A., Ross, F.A., Gowans, G.J., Tibarewal, P., Leslie, N.R., Hardie, D.G.: Phosphorylation by akt within the st loop of ampk-α1 down-regulates its activation in tumour cells. The Biochemical journal **459**, 275–87 (2014)

Hardie, D.G., Ross, F.A., Hawley, S.A.: Ampk: a nutrient and energy sensor that maintains energy homeostasis. Nature reviews. Molecular cell biology **13**, 251–62 (2012)

Hwang, J.-w., Rajendrasozhan, S., Yao, H., Chung, S., Sundar, I.K., Huyck, H.L., Pryhuber, G.S., Kinnula, V.L., Rahman, I.: Foxo3 deficiency leads to increased susceptibility to cigarette smoke-induced inflammation, airspace enlargement, and chronic obstructive pulmonary disease. Journal of immunology (Baltimore, Md. : 1950) **187**, 987–98 (2011)

He, G., Zhang, Y.-W., Lee, J.-H., Zeng, S.X., Wang, Y.V., Luo, Z., Dong, X.C., Viollet, B., Wahl, G.M., Lu, H.: Amp-activated protein kinase induces p53 by phosphorylating mdmx and inhibiting its activity. Molecular and cellular biology **34**, 148–57 (2014)

Hu, W., Zhang, C., Wu, R., Sun, Y., Levine, A., Feng, Z.: Glutaminase 2, a novel p53 target gene regulating energy metabolism and antioxidant function. Proceedings of the National Academy of Sciences of the United States of America **107**, 7455–60 (2010)

Jiang, M., Kolehmainen, P., Kakkola, L., Maljanen, S., Melén, K., Smura, T., Julkunen, I., Österlund, P.: Sars-cov-2 isolates show impaired replication in human immune cells but differential ability to replicate and induce innate immunity in lung epithelial cells. Microbiology spectrum **9**, 0077421 (2021)

Jung, I.L., Kang, H.J., Kim, K.C., Kim, I.G.: Pten/pakt/p53 signaling pathway correlates with the radioresponse of non-small cell lung cancer. International journal of molecular medicine **25**, 517–23 (2010)

Jafarzadeh, A., Nemati, M., Jafarzadeh, S.: Contribution of stat3 to the pathogenesis of covid-19. Microbial pathogenesis **154**, 104836 (2021)

Jyonouchi, H.: Airway epithelium and apoptosis. Apoptosis : an international journal on programmed cell death **4**, 407–17 (1999)

Jiang, S., Yang, H., Sun, Z., Zhang, Y., Li, Y., Li, J.: The basis of complications in the context of sars-cov-2 infection: Pathological activation of adam17. Biochemical and Biophysical Research Communications **679**, 37–46 (2023)

Koumenis, C., Alarcon, R., Hammond, E., Sutphin, P., Hoffman, W., Murphy, M., Derr, J., Taya, Y., Lowe, S.W., Kastan, M., Giaccia, A.: Regulation of

p53 by hypoxia: dissociation of transcriptional repression and apoptosis from p53-dependent transactivation. Molecular and cellular biology **21**, 1297–310 (2001)

Kanehisa Laboratories: KEGG: Kyoto Encyclopedia of Genes and Genomes. https://www.kegg.jp/. Accessed: 2025-09-04

Klemm, C., Bruchhagen, C., Krüchten, A., Niemann, S., Löffler, B., Peters, G., Ludwig, S., Ehrhardt, C.: Mitogen-activated protein kinases (mapks) regulate il-6 over-production during concomitant influenza virus and staphylococcus aureus infection. Scientific reports **7**, 42473 (2017)

Kazyken, D., Magnuson, B., Bodur, C., Acosta-Jaquez, H.A., Zhang, D., Tong, X., Barnes, T.M., Steinl, G.K., Patterson, N.E., Altheim, C.H., Sharma, N., Inoki, K., Cartee, G.D., Bridges, D., Yin, L., Riddle, S.M., Fingar, D.C.: Ampk directly activates mtorc2 to promote cell survival during acute energetic stress. Science signaling **12**, 3249 (2019)

Koundouros, N., Poulogiannis, G.: Phosphoinositide 3-kinase/akt signaling and redox metabolism in cancer. Frontiers in oncology **8**, 160 (2018)

Kucharczak, J., Simmons, M.J., Fan, Y., Gélinas, C.: To be, or not to be: Nf-kappab is the answer–role of rel/nf-kappab in the regulation of apoptosis. Oncogene **22**, 8961–82 (2003)

Khan, S., Shafiei, M.S., Longoria, C., Schoggins, J.W., Savani, R.C., Zaki, H.: Sars-cov-2 spike protein induces inflammation via tlr2-dependent activation of the nf-κb pathway. eLife **10**, 68563 (2021)

Kaminskyy, V.O., Zhivotovsky, B.: Free radicals in cross talk between autophagy and apoptosis. Antioxidants & redox signaling **21**, 86–102 (2014)

Krześniak, M., Zajkowicz, A., Matuszczyk, I., Rusin, M.: Rapamycin prevents strong phosphorylation of p53 on serine 46 and attenuates activation of the p53 pathway in a549 lung cancer cells exposed to actinomycin d. Mechanisms of ageing and development **139**, 11–21 (2014)

Lin, C.-H., Hong, Y.-C., Kao, S.-H.: Aeroallergen der p 2 induces apoptosis of bronchial epithelial beas-2b cells via activation of both intrinsic and extrinsic pathway. Cell & bioscience **5**, 71 (2015)

Ling, N.X.Y., Kaczmarek, A., Hoque, A., Davie, E., Ngoei, K.R.W., Morrison, K.R., Smiles, W.J., Forte, G.M., Wang, T., Lie, S., Dite, T.A., Langendorf, C.G., Scott, J.W., Oakhill, J.S., Petersen, J.: mtorc1 directly inhibits ampk to promote cell proliferation under nutrient stress. Nature metabolism **2**, 41–49 (2020)

Li, Y., Liang, R., Sun, M., Li, Z., Sheng, H., Wang, J., Xu, P., Liu, S., Yang, W., Lu, B., Zhang, S., Shan, C.: Ampk-dependent phosphorylation of hdac8 triggers pgm1

expression to promote lung cancer cell survival under glucose starvation. Cancer letters **478**, 82–92 (2020)

Laird, M.H.W., Rhee, S.H., Perkins, D.J., Medvedev, A.E., Piao, W., Fenton, M.J., Vogel, S.N.: Tlr4/myd88/pi3k interactions regulate tlr4 signaling. Journal of leukocyte biology **85**, 966–77 (2009)

Liu, J., Uematsu, H., Tsuchida, N., Ikeda, M.-A.: Essential role of caspase-8 in p53/p73-dependent apoptosis induced by etoposide in head and neck carcinoma cells. Molecular cancer **10**, 95 (2011)

Li, L., Wu, W., Huang, W., Hu, G., Yuan, W., Li, W.: Nf-κb rnai decreases the bax/bcl-2 ratio and inhibits tnf-α-induced apoptosis in human alveolar epithelial cells. Inflammation research : official journal of the European Histamine Research Society ... [et al.] **62**, 387–97 (2013)

Li, S.-W., Wang, C.-Y., Jou, Y.-J., Yang, T.-C., Huang, S.-H., Wan, L., Lin, Y.-J., Lin, C.-W.: Sars coronavirus papain-like protease induces egr-1-dependent up-regulation of tgf-β1 via ros/p38 mapk/stat3 pathway. Scientific reports **6**, 25754 (2016)

Lee, H.-Y., Youn, S.-W., Kim, J.-Y., Park, K.-W., Hwang, C.-I., Park, W.-Y., Oh, B.-H., Park, Y.-B., Walsh, K., Seo, J.-S., Kim, H.-S.: Foxo3a turns the tumor necrosis factor receptor signaling towards apoptosis through reciprocal regulation of c-jun n-terminal kinase and nf-kappab. Arteriosclerosis, thrombosis, and vascular biology **28**, 112–20 (2008)

Li, S., Zhang, Y., Guan, Z., Li, H., Ye, M., Chen, X., Shen, J., Zhou, Y., Shi, Z.-L., Zhou, P., Peng, K.: Sars-cov-2 triggers inflammatory responses and cell death through caspase-8 activation. Signal transduction and targeted therapy **5**, 235 (2020)

Labuschagne, C.F., Zani, F., Vousden, K.H.: Control of metabolism by p53 - cancer and beyond. Biochimica et biophysica acta. Reviews on cancer **1870**, 32–42 (2018)

Magro, G.: Sars-cov-2 and covid-19: Is interleukin-6 (il-6) the 'culprit lesion' of ards onset? what is there besides tocilizumab? sgp130fc. Cytokine: X **2**, 100029 (2020)

Marzi, L., Combes, E., Vié, N., Ayrolles-Torro, A., Tosi, D., Desigaud, D., Perez-Gracia, E., Larbouret, C., Montagut, C., Iglesias, M., Jarlier, M., Denis, V., Linares, L.K., Lam, E.W.-F., Martineau, P., Del Rio, M., Gongora, C.: Foxo3a and the mapk p38 are activated by cetuximab to induce cell death and inhibit cell proliferation and their expression predicts cetuximab efficacy in colorectal cancer. British journal of cancer **115**, 1223–1233 (2016)

Matsuyama, T., Kubli, S.P., Yoshinaga, S.K., Pfeffer, K., Mak, T.W.: An aberrant stat pathway is central to covid-19. Cell death and differentiation **27**, 3209–3225 (2020)

Ma-Lauer, Y., Carbajo-Lozoya, J., Hein, M.Y., Müller, M.A., Deng, W., Lei, J., Meyer, B., Kusov, Y., Brunn, B., Bairad, D.R., Hünten, S., Drosten, C., Hermeking, H., Leonhardt, H., Mann, M., Hilgenfeld, R., Brunn, A.: p53 down-regulates sars coronavirus replication and is targeted by the sars-unique domain and plpro via e3 ubiquitin ligase rchy1. Proceedings of the National Academy of Sciences of the United States of America **113**, 5192–201 (2016)

Micheau, O., Lens, S., Gaide, O., Alevizopoulos, K., Tschopp, J.: Nf-kappab signals induce the expression of c-flip. Molecular and cellular biology **21**, 5299–305 (2001)

Mazewski, C., Perez, R.E., Fish, E.N., Platanias, L.C.: Type i interferon (ifn)-regulated activation of canonical and non-canonical signaling pathways. Frontiers in immunology **11**, 606456 (2020)

Mattos Pereira, V., Thakar, A., Nair, S.: Targeting irhom2/adam17 attenuates covid-19-induced cytokine release from cultured lung epithelial cells. Biochemistry and biophysics reports **39**, 101811 (2024)

Martin-Sancho, L., Lewinski, M.K., Pache, L., Stoneham, C.A., Yin, X., Becker, M.E., Pratt, D., Churas, C., Rosenthal, S.B., Liu, S., Weston, S., De Jesus, P.D., O'Neill, A.M., Gounder, A.P., Nguyen, C., Pu, Y., Curry, H.M., Oom, A.L., Miorin, L., Rodriguez-Frandsen, A., Zheng, F., Wu, C., Xiong, Y., Urbanowski, M., Shaw, M.L., Chang, M.W., Benner, C., Hope, T.J., Frieman, M.B., García-Sastre, A., Ideker, T., Hultquist, J.F., Guatelli, J., Chanda, S.K.: Functional landscape of sars-cov-2 cellular restriction. Molecular cell **81**, 2656–26688 (2021)

Mokuda, S., Tokunaga, T., Masumoto, J., Sugiyama, E.: Angiotensin-converting enzyme 2, a sars-cov-2 receptor, is upregulated by interleukin 6 through stat3 signaling in synovial tissues. The Journal of Rheumatology **47**(10), 1593–1595 (2020)

Mukherjee, S.: Toll-like receptor 4 in covid-19: friend or foe? Future virology, 10–221720210249 (2022)

Mohd Zawawi, Z., Kalyanasundram, J., Mohd Zain, R., Thayan, R., Basri, D.F., Yap, W.B.: Prospective roles of tumor necrosis factor-alpha (tnf-α) in covid-19: Prognosis, therapeutic and management. International journal of molecular sciences **24**, 6142 (2023)

Ni, W., Yang, X., Yang, D., Bao, J., Li, R., Xiao, Y., Hou, C., Wang, H., Liu, J., Yang, D., Xu, Y., Cao, Z., Gao, Z.: Role of angiotensin-converting enzyme 2 (ace2) in covid-19. Critical care (London, England) **24**, 422 (2020)

Olímpio, F., Andreata-Santos, R., Rosa, P.C., Santos, W., Oliveira, C., Aimbire, F.: Lactobacillus rhamnosus restores antiviral signaling and attenuates cytokines secretion from human bronchial epithelial cells exposed to cigarette smoke and infected with sars-cov-2. Probiotics and antimicrobial proteins **15**, 1513–1528 (2023)

Okoh, V.O., Felty, Q., Parkash, J., Poppiti, R., Roy, D.: Reactive oxygen species via redox signaling to pi3k/akt pathway contribute to the malignant growth of 4-hydroxy estradiol-transformed mammary epithelial cells. PloS one **8**, 54206 (2013)

Ooppachai, C., Limtrakul Dejkriengkraikul, P., Yodkeeree, S.: Dicentrine potentiates tnf-α-induced apoptosis and suppresses invasion of a549 lung adenocarcinoma cells via modulation of nf-κb and ap-1 activation. Molecules (Basel, Switzerland) **24**, 4100 (2019)

Park, S.H., Choi, H.-J., Lee, S.Y., Han, J.-S.: Tlr4-mediated irak1 activation induces tnf-α expression via jnk-dependent nf-κb activation in human bronchial epithelial cells. European Journal of Inflammation **13**(3), 183–195 (2015)

Püschel, F., Favaro, F., Redondo-Pedraza, J., Lucendo, E., Iurlaro, R., Marchetti, S., Majem, B., Eldering, E., Nadal, E., Ricci, J.-E., Chevet, E., Muñoz-Pinedo, C.: Starvation and antimetabolic therapy promote cytokine release and recruitment of immune cells. Proceedings of the National Academy of Sciences of the United States of America **117**, 9932–9941 (2020)

Porter, A.G., Jänicke, R.U.: Emerging roles of caspase-3 in apoptosis. Cell death and differentiation **6**, 99–104 (1999)

Platanias, L.C.: Mechanisms of type-i- and type-ii-interferon-mediated signalling. Nature reviews. Immunology **5**, 375–86 (2005)

Papp, M., Li, X., Zhuang, J., Wang, R., Uhal, B.D.: Angiotensin receptor subtype at(1) mediates alveolar epithelial cell apoptosis in response to ang ii. American journal of physiology. Lung cellular and molecular physiology **282**, 713–8 (2002)

Patra, T., Meyer, K., Geerling, L., Isbell, T.S., Hoft, D.F., Brien, J., Pinto, A.K., Ray, R.B., Ray, R.: Sars-cov-2 spike protein promotes il-6 trans-signaling by activation of angiotensin ii receptor signaling in epithelial cells. PLoS pathogens **16**, 1009128 (2020)

Polke, M., Seiler, F., Lepper, P.M., Kamyschnikow, A., Langer, F., Monz, D., Herr, C., Bals, R., Beisswenger, C.: Hypoxia and the hypoxia-regulated transcription factor hif-1α suppress the host defence of airway epithelial cells. Innate immunity **23**, 373–380 (2017)

Qian, S., Wei, Z., Yang, W., Huang, J., Yang, Y., Wang, J.: The role of bcl-2 family proteins in regulating apoptosis and cancer therapy. Frontiers in oncology **12**, 985363 (2022)

Qiu, Q., Yang, Z., Cao, F., Yang, C., Hardy, P., Yan, X., Yang, S., Xiong, W.: Activation of nlrp3 inflammasome by lymphocytic microparticles via tlr4 pathway contributes to airway inflammation. Experimental cell research **386**, 111737 (2020)

Rodriguez-Perez, A.I., Labandeira, C.M., Pedrosa, M.A., Valenzuela, R., Suarez-Quintanilla, J.A., Cortes-Ayaso, M., Mayán-Conesa, P., Labandeira-Garcia, J.L.: Autoantibodies against ace2 and angiotensin type-1 receptors increase severity of covid-19. Journal of autoimmunity **122**, 102683 (2021)

Ramasamy, S., Subbian, S.: Critical determinants of cytokine storm and type i interferon response in covid-19 pathogenesis. Clinical microbiology reviews **34**, 00299–20 (2021)

Riethmueller, S., Somasundaram, P., Ehlers, J.C., Hung, C.-W., Flynn, C.M., Lokau, J., Agthe, M., Düsterhöft, S., Zhu, Y., Grötzinger, J., *et al.*: Proteolytic origin of the soluble human il-6r in vivo and a decisive role of n-glycosylation. PLoS biology **15**(1), 2000080 (2017)

Rebendenne, A., Valadão, A.L.C., Tauziet, M., Maarifi, G., Bonaventure, B., McKellar, J., Planès, R., Nisole, S., Arnaud-Arnould, M., Moncorgé, O., Goujon, C.: Sars-cov-2 triggers an mda-5-dependent interferon response which is unable to control replication in lung epithelial cells. Journal of virology **95**, 02415–20 (2021)

Safa, A.R.: Roles of c-flip in apoptosis, necroptosis, and autophagy. Journal of carcinogenesis & mutagenesis **Suppl 6**, 003 (2013)

Stolarczyk, M., Amatngalim, G.D., Yu, X., Veltman, M., Hiemstra, P.S., Scholte, B.J.: Adam 17 and egfr regulate il-6 receptor and amphiregulin mrna expression and release in cigarette smoke-exposed primary bronchial epithelial cells from patients with chronic obstructive pulmonary disease (copd). Physiological reports **4**(16), 12878 (2016)

Saccon, E., Chen, X., Mikaeloff, F., Rodriguez, J.E., Szekely, L., Vinhas, B.S., Krishnan, S., Byrareddy, S.N., Frisan, T., Vegvari, A., Mirazimi, A., Neogi, U., Gupta, S.: Cell-type-resolved quantitative proteomics map of interferon response against sars-cov-2. iScience **24**, 102420 (2021)

Schaub, M.A., Henzinger, T.A., Fisher, J.: Qualitative networks: a symbolic approach to analyze biological signaling networks. BMC systems biology **1**, 4 (2007)

Shin, H.J., Jo, M.J., Jin, I.S., Park, C.-W., Kim, J.-S., Shin, D.H.: Optimization and pharmacokinetic evaluation of synergistic fenbendazole and rapamycin co-encapsulated in methoxy poly(ethylene glycol)-¡i¿b¡/i¿-poly(caprolactone) polymeric micelles. International journal of nanomedicine **16**, 4873–4889 (2021)

Sawada, M., Kiyono, T., Nakashima, S., Shinoda, J., Naganawa, T., Hara, S., Iwama, T., Sakai, N.: Molecular mechanisms of tnf-alpha-induced ceramide formation in human glioma cells: P53-mediated oxidant stress-dependent and -independent pathways. Cell death and differentiation **11**, 997–1008 (2004)

Sikder, M.A., Lee, H.J., Mia, M.Z., Park, S.H., Ryu, J., Kim, J.-H., Min, S.Y., Hong,

J.-H., Seok, J.H., Lee, C.J.: Inhibition of tnf-α-induced muc5ac mucin gene expression and production by wogonin through the inactivation of nf-κb signaling in airway epithelial cells. Phytotherapy research : PTR **28**, 62–8 (2014)

Skurk, C., Maatz, H., Kim, H.-S., Yang, J., Abid, M.R., Aird, W.C., Walsh, K.: The akt-regulated forkhead transcription factor foxo3a controls endothelial cell viability through modulation of the caspase-8 inhibitor flip. The Journal of biological chemistry **279**, 1513–25 (2004)

Sodeifian, F., Nikfarjam, M., Kian, N., Mohamed, K., Rezaei, N.: The role of type i interferon in the treatment of covid-19. Journal of medical virology **94**, 63–81 (2022)

Sermeus, A., Rebucci, M., Fransolet, M., Flamant, L., Desmet, D., Delaive, E., Arnould, T., Michiels, C.: Differential effect of hypoxia on etoposide-induced dna damage response and p53 regulation in different cell types. Journal of cellular physiology **228**, 2365–76 (2013)

Seo, H.-S., Sikder, M.A., Lee, H.J., Ryu, J., Lee, C.J.: Apigenin inhibits tumor necrosis factor-α-induced production and gene expression of mucin through regulating nuclear factor-kappa b signaling pathway in airway epithelial cells. Biomolecules & therapeutics **22**, 525–31 (2014)

Stepanova, G.: Biologia futura: is adam 17 the reason for covid-19 susceptibility in hyperglycemic and diabetic patients? Biologia futura **72**, 291–297 (2021)

Suzuki, S., Tanaka, T., Poyurovsky, M.V., Nagano, H., Mayama, T., Ohkubo, S., Lokshin, M., Hosokawa, H., Nakayama, T., Suzuki, Y., Sugano, S., Sato, E., Nagao, T., Yokote, K., Tatsuno, I., Prives, C.: Phosphate-activated glutaminase (gls2), a p53-inducible regulator of glutamine metabolism and reactive oxygen species. Proceedings of the National Academy of Sciences of the United States of America **107**, 7461–6 (2010)

Salman, A.A., Waheed, M.H., Ali-Abdulsahib, A.A., Atwan, Z.W.: Low type i interferon response in covid-19 patients: Interferon response may be a potential treatment for covid-19. Biomedical reports **14**, 43 (2021)

Suhail, S., Zajac, J., Fossum, C., Lowater, H., McCracken, C., Severson, N., Laatsch, B., Narkiewicz-Jodko, A., Johnson, B., Liebau, J., Bhattacharyya, S., Hati, S.: Role of oxidative stress on sars-cov (sars) and sars-cov-2 (covid-19) infection: A review. The protein journal **39**, 644–656 (2020)

Tolomeo, M., Cavalli, A., Cascio, A.: Stat1 and its crucial role in the control of viral infections. International journal of molecular sciences **23**, 4095 (2022)

COVID-19 Disease Map Community, Ostaszewski, M., Niarakis, A., Mazein, A., Kuperstein, I., Phair, R., Orta-Resendiz, A., Singh, V., Aghamiri, S.S., Acencio, M.L., Glaab, E., Ruepp, A., Fobo, G., Montrone, C., Brauner, B., Frishman, G.,

Gómez, L.C.M., Somers, J., Hoch, M., Gupta, S.K., Scheel, J., Borlinghaus, H., Czauderna, T., Schreiber, F., Montagud, A., Leon, M.P., Funahashi, A., Hiki, Y., Hiroi, N., Yamada, T.G., Dräger, A., Renz, A., Naveez, M., Bocskei, Z., Messina, F., Börnigen, D., Fergusson, L., Conti, M., Rameil, M., Nakonecnij, V., Vanhoefer, J., Schmiester, L., Wang, M., Ackerman, E.E., Shoemaker, J.E., Zucker, J., Oxford, K., Teuton, J., Kocakaya, E., Waard, A.: Covid19 disease map, a computational knowledge repository of virus–host interaction mechanisms. Molecular Systems Biology **17**(10), 10387 (2021)

Uliczka, K., Bossen, J., Zissler, U.M., Fink, C., Niu, X., Pieper, M., Prange, R.D., Vock, C., Wagner, C., Knop, M., et al.: Foxo factors are essential for maintaining organ homeostasis by acting as stress sensors in airway epithelial cells. bioRxiv, 2024–01 (2024)

Ushijima, H., Suzuki, Y., Oike, T., Komachi, M., Yoshimoto, Y., Ando, K., Okonogi, N., Sato, H., Noda, S.-E., Saito, J.-I., Nakano, T.: Radio-sensitization effect of an mtor inhibitor, temsirolimus, on lung adenocarcinoma a549 cells under normoxic and hypoxic conditions. Journal of radiation research **56**, 663–8 (2015)

Valentine, R.J., Coughlan, K.A., Ruderman, N.B., Saha, A.K.: Insulin inhibits ampk activity and phosphorylates ampk ser$^{485}/^{491}$ through akt in hepatocytes, myotubes and incubated rat skeletal muscle. Archives of biochemistry and biophysics **562**, 62–9 (2014)

Vollmer, S., Strickson, S., Zhang, T., Gray, N., Lee, K.L., Rao, V.R., Cohen, P.: The mechanism of activation of irak1 and irak4 by interleukin-1 and toll-like receptor agonists. The Biochemical journal **474**, 2027–2038 (2017)

Weinlich, R., Green, D.R.: The two faces of receptor interacting protein kinase-1. Molecular cell **56**, 469–80 (2014)

Wang, X., Ma, J., Lin, D., Bai, Y., Zhang, D., Jia, X., Gao, J.: Mir-145-5p reduced ang ii-induced ace2 shedding and the inflammatory response in alveolar epithelial cells by targeting adam17 and inhibiting the at1r/adam17 pathway. European journal of pharmacology **971**, 176392 (2024)

Wong, C.K., Wang, C.B., Ip, W.K., Tian, Y.P., Lam, C.W.K.: Role of p38 mapk and nf-kb for chemokine release in coculture of human eosinophils and bronchial epithelial cells. Clinical and experimental immunology **139**, 90–100 (2005)

Wei, H., Wang, H., Wang, G., Qu, L., Jiang, L., Dai, S., Chen, X., Zhang, Y., Chen, Z., Li, Y., Guo, M., Chen, Y.: Structures of p53/bcl-2 complex suggest a mechanism for p53 to antagonize bcl-2 activity. Nature communications **14**, 4300 (2023)

Wang, W., Weng, J., Yu, L., Huang, Q., Jiang, Y., Guo, X.: Role of tlr4-p38 mapk-hsp27 signal pathway in lps-induced pulmonary epithelial hyperpermeability. BMC pulmonary medicine **18**, 178 (2018)

Wang, K., Xuan, Z., Liu, X., Zheng, M., Yang, C., Wang, H.: Immunomodulatory role of metalloproteinase adam17 in tumor development. Frontiers in immunology **13**, 1059376 (2022)

Xing, Z., Harper, R., Anunciacion, J., Yang, Z., Gao, W., Qu, B., Guan, Y., Cardona, C.J.: Host immune and apoptotic responses to avian influenza virus h9n2 in human tracheobronchial epithelial cells. American journal of respiratory cell and molecular biology **44**, 24–33 (2011)

Yadav, S., Kalra, N., Ganju, L., Singh, M.: Activator protein-1 (ap-1): a bridge between life and death in lung epithelial (a549) cells under hypoxia. Molecular and cellular biochemistry **436**, 99–110 (2017)

Yamada, T., Sato, S., Sotoyama, Y., Orba, Y., Sawa, H., Yamauchi, H., Sasaki, M., Takaoka, A.: Rig-i triggers a signaling-abortive anti-sars-cov-2 defense in human lung cells. Nature immunology **22**, 820–828 (2021)

Yang, Q., Tang, J., Cao, J., Liu, F., Fu, M., Xue, B., Zhou, A., Chen, S., Liu, J., Zhou, Y., Shi, Y., Peng, W., Chen, X.: Sars-cov-2 infection activates creb/cbp in cellular cyclic amp-dependent pathways. Journal of medical virology **95**, 28383 (2023)

Zhang, Y., Gargan, S., Roche, F.M., Frieman, M., Stevenson, N.J.: Inhibition of the ifn-α jak/stat pathway by mers-cov and sars-cov-1 proteins in human epithelial cells. Viruses **14**, 667 (2022)

Zarubin, T., Han, J.: Activation and signaling of the p38 map kinase pathway. Cell research **15**, 11–8 (2005)

Zhao, Y., Hu, X., Liu, Y., Dong, S., Wen, Z., He, W., Zhang, S., Huang, Q., Shi, M.: Ros signaling under metabolic stress: cross-talk between ampk and akt pathway. Molecular cancer **16**, 79 (2017)

Ziello, J.E., Jovin, I.S., Huang, Y.: Hypoxia-inducible factor (hif)-1 regulatory pathway and its potential for therapeutic intervention in malignancy and ischemia. The Yale journal of biology and medicine **80**, 51–60 (2007)

Zhao, Y., Liu, Y.-S.: Longevity factor foxo3: A key regulator in aging-related vascular diseases. Frontiers in cardiovascular medicine **8**, 778674 (2021)

Zegeye, M.M., Lindkvist, M., Fälker, K., Kumawat, A.K., Paramel, G., Grenegård, M., Sirsjö, A., Ljungberg, L.U.: Activation of the jak/stat3 and pi3k/akt pathways are crucial for il-6 trans-signaling-mediated pro-inflammatory response in human vascular endothelial cells. Cell communication and signaling : CCS **16**, 55 (2018)

Zhang, C., Lin, M., Wu, R., Wang, X., Yang, B., Levine, A.J., Hu, W., Feng, Z.: Parkin, a p53 target gene, mediates the role of p53 in glucose metabolism and the warburg effect. Proceedings of the National Academy of Sciences of the United

States of America **108**, 16259–64 (2011)

Zhang, C., Liu, J., Wang, J., Zhang, T., Xu, D., Hu, W., Feng, Z.: The interplay between tumor suppressor p53 and hypoxia signaling pathways in cancer. Frontiers in cell and developmental biology **9**, 648808 (2021)

Zheng, F., Tang, Q., Wu, J., Zhao, S., Liang, Z., Li, L., Wu, W., Hann, S.: p38α mapk-mediated induction and interaction of foxo3a and p53 contribute to the inhibited-growth and induced-apoptosis of human lung adenocarcinoma cells by berberine. Journal of experimental & clinical cancer research : CR **33**, 36 (2014)

Zhou, Y., Wang, M., Li, Y., Wang, P., Zhao, P., Yang, Z., Wang, S., Zhang, L., Li, Z., Jia, K., Zhong, C., Li, N., Yu, Y., Hou, J.: Sars-cov-2 spike protein enhances ace2 expression via facilitating interferon effects in bronchial epithelium. Immunology letters **237**, 33–41 (2021)

Zhang, X., Wu, K., Wang, D., Yue, X., Song, D., Zhu, Y., Wu, J.: Nucleocapsid protein of sars-cov activates interleukin-6 expression through cellular transcription factor nf-kappab. Virology **365**, 324–35 (2007)

Zhu, Z., Zhou, H., Chen, F., Deng, J., Yin, L., He, B., Hu, Q., Wang, T.: Sinomenine derivatives: Synthesis, antitumor activity, and apoptotic induction in mcf-7 cells via il-6/pi3k/akt/nf-κb signaling pathway. ChemMedChem **17**, 202200234 (2022)

Zhu, Z., Zheng, Z., Liu, J.: Comparison of covid-19 and lung cancer ¡i¿via¡/i¿ reactive oxygen species signaling. Frontiers in oncology **11**, 708263 (2021)
